# Cardiovascular Protective Effects of NP-6A4, a Drug with the FDA Designation for Pediatric Cardiomyopathy, in Female Rats with Obesity and Pre-Diabetes

**DOI:** 10.3390/cells12101373

**Published:** 2023-05-12

**Authors:** Anthony M. Belenchia, Asma Boukhalfa, Vincent G. DeMarco, Alexander Mehm, Abuzar Mahmood, Pei Liu, Yinian Tang, Madhavi P. Gavini, Brian Mooney, Howard H. Chen, Lakshmi Pulakat

**Affiliations:** 1 Dalton Cardiovascular Research Center and Department of Nutrition and Exercise Physiology, University of Missouri, Columbia, MO 65211, USA; 2 Molecular Cardiology Research Institute, Tufts Medical Center, and Department of Medicine, Tufts University, Boston, MA 02111, USA; 3 Department of Medicine, University of Missouri, Columbia, MO 65212, USA; 4 Department of Neuroscience, Brandeis University, Waltham, MA 02453, USA; 5 Charles W. Gehrke Proteomics Center, University of Missouri, Columbia, MO 65211, USA; 6 Novopyxis Inc., Boston, MA 02108, USA; 7 Division of Biochemistry, University of Missouri, Columbia, MO 65211, USA

**Keywords:** cardiovascular disease (CVD), body mass index (BMI), Angiotensin II receptor AT2 (AT2R), Zucker diabetic fatty-female (ZDF-F), Zucker diabetic fatty-male (ZDF-M), Zucker lean-female (ZL-F), Zucker lean-male (ZL-M), obesity

## Abstract

Background: Obese and pre-diabetic women have a higher risk for cardiovascular death than age-matched men with the same symptoms, and there are no effective treatments. We reported that obese and pre-diabetic female Zucker Diabetic Fatty (ZDF-F) rats recapitulate metabolic and cardiac pathology of young obese and pre-diabetic women and exhibit suppression of cardio-reparative AT2R. Here, we investigated whether NP-6A4, a new AT2R agonist with the FDA designation for pediatric cardiomyopathy, mitigate heart disease in ZDF-F rats by restoring AT2R expression. Methods: ZDF-F rats on a high-fat diet (to induce hyperglycemia) were treated with saline, NP-6A4 (10 mg/kg/day), or NP-6A4 + PD123319 (AT2R-specific antagonist, 5 mg/kg/day) for 4 weeks (n = 21). Cardiac functions, structure, and signaling were assessed by echocardiography, histology, immunohistochemistry, immunoblotting, and cardiac proteome analysis. Results: NP-6A4 treatment attenuated cardiac dysfunction, microvascular damage (−625%) and cardiomyocyte hypertrophy (−263%), and increased capillary density (200%) and AT2R expression (240%) (*p* < 0.05). NP-6A4 activated a new 8-protein autophagy network and increased autophagy marker LC3-II but suppressed autophagy receptor p62 and autophagy inhibitor Rubicon. Co-treatment with AT2R antagonist PD123319 suppressed NP-6A4’s protective effects, confirming that NP-6A4 acts through AT2R. NP-6A4-AT2R-induced cardioprotection was independent of changes in body weight, hyperglycemia, hyperinsulinemia, or blood pressure. Conclusions: Cardiac autophagy impairment underlies heart disease induced by obesity and pre-diabetes, and there are no drugs to re-activate autophagy. We propose that NP-6A4 can be an effective drug to reactivate cardiac autophagy and treat obesity- and pre-diabetes-induced heart disease, particularly for young and obese women.

## 1. Introduction

Ischemic heart disease is the number one killer of both men and women worldwide [1,2]. Obesity, a complex metabolic disorder that afflicts 35% of the adult population in the United States is a critical contributor to Type 2 Diabetes Mellitus (T2DM), ischemic heart disease, and cardiovascular death [1,2,3,4,5]. Clinical and epidemiological evidence suggests that obesity- and diabetes-induced reduction in cardiac capillary density (microvascular rarefaction) and increased vascular-endothelial damage contribute to impaired myocardial blood flow that causes ischemia, cardiac dysfunction, and heart failure [6,7,8]. Notably, data from the Framingham Heart Study shows that obesity increases ischemic heart disease in men by 46% and in women by 64%—further highlighting the disproportionally higher risk of cardiovascular death in obese women compared to obese men [9]. Epidemiological and clinical data suggest that healthy pre-menopausal women have a 10-year advantage in developing cardiovascular disease compared to age-matched healthy men. However, obesity and T2DM clinically attenuate this female-specific advantage in cardiovascular risk [9,10,11,12]. An increase in body mass index (BMI) increases the hazard ratio for acute myocardial infarction (AMI), ischemic stroke (IS), and death due to cardiovascular disease (CVD) in young women [11]. A recent meta-analysis involving 5,162,654 participants shows that women with T2DM had a 58% greater risk of coronary heart disease (CHD) mortality compared to men with the same condition [13]. Diabetic women are also shown to have a similar risk for atherosclerotic cardiovascular disease (ASCVD) as diabetic men and do not have the female-specific protection form ASCVD [14]. Moreover, young obese and diabetic women have additional cardiovascular risk factors, related to complications of pregnancy and childbirth, and effective cardiovascular protective treatments, such as renin-angiotensin-aldosterone system (RAAS) blockers, are often contra-indicated in this highly vulnerable population [15].

Autophagy is a lysosomal degradation process that removes misfolded proteins and damaged organelles and plays an important role in maintaining cellular metabolic homeostasis and cardiac health [16,17,18]. Although reactivating autophagy has been proposed by many researchers as an effective treatment to mitigate obesity- and T2DM-induced microvascular damage and cardiomyopathy that leads to death [19,20,21], there are no treatments in the clinic to safely activate cardiac autophagy in obese and diabetic patients. Rapamycin (Rap), an inhibitor of mammalian target for rapamycin complexes 1 and 2 (mTORC1 and mTORC2), is an effective autophagy inducer in many cell types [21]. However, research shows that Rap-induced mTORC1 and mTORC2 inhibition causes an excessive autophagic response resulting in functional impairment of human endothelial cells, reduces endothelial nitric oxide synthase levels, suppresses nitric oxide production that causes vascular-endothelial uncoupling, and reduces human endothelial cell viability [22,23]. Moreover, even at a low dose (0.2 mg/kg), rapamycin induces excessive autophagy that impairs pancreatic β cells, resulting in de novo diabetes in pre-clinical models [24]. Thus, developing new therapeutic approaches to induce cardioprotective autophagy without damaging endothelial cell function and viability to protect the human heart is a critical unmet medical need for obese and diabetic patients.

We reported previously that young, obese Zucker Diabetic Fatty (ZDF) female (ZDF-F) rats fed a high-fat diet recapitulate the metabolic and cardiac pathology of young obese women [25]. Unlike male ZDF (ZDF-M) rats that develop hyperphagia and hyperglycemia on normal chow, ZDF-F rats do not develop hyperglycemia on normal chow; although, they do develop hyperphagia and obesity. In this context, ZDF-F rats are similar to obese women who are also more resistant to hyperglycemia than age-matched obese men [25,26]. However, ZDF-F rats can develop hyperglycemia when fed with high-fat chow (Research Diet #D12468). Similar to obese women, these obese ZDF-F rats fed with a high-fat diet have higher body fat and lower muscle mass than age-matched ZDF-M rats and healthy Zucker lean (ZL) female (ZL-F) rats [25]. Therefore, we used ZDF-F rats fed with a high-fat diet to mimic the metabolic phenotype similar to obese women with pre-diabetes.

ZDF rats of both sexes used in our study (ZDF-F rats fed with D12468 and ZDF-M rats on normal chow) exhibit cardiac microvascular rarefaction and diastolic and systolic dysfunction compared to sex- and age-matched healthy ZL rats [25]. However, ZDF-F rats develop cardiac scar tissues characterized by cardiomyocyte loss indicating more severe cardiac structural damage [25]. Our analysis of cardiac biomarkers associated with sex differences in cardiac physiology identified that cardiac expression of Angiotensin II receptor AT2R, a cardiovascular protective molecule, exhibits sexual dimorphism in healthy ZL rats [25]. The ZL-F rats have increased cardiac expression of AT2R (increased by 188%) compared to age-matched ZL-M rats [25]. However, young and obese ZDF-F rats do not have this cardioprotective AT2R advantage since their cardiac AT2R expression is suppressed (−238%) compared to healthy ZL-F rats [25]. How AT2R regulates obesity-induced cardiac dysfunction and microvascular damage is not elucidated. Therefore, we performed studies to uncover how NP-6A4 [27,28,29], a peptide agonist of AT2R, modulates cardiac structural and functional damage in young obese and hyperglycemic ZDF-F rats.

We chose NP-6A4 to induce new AT2R signaling because it has been shown to be more effective in protecting mouse cardiomyocyte HL-1 cells and human coronary artery vascular smooth muscle cells (hCAVSMCs) from acute nutrient deficiency than widely used β-adrenergic receptor blockers, AT1 receptor blocker losartan, and another AT2R agonist CGP42112A, as we reported previously [27]. NP-6A4 has an orphan drug designation from the FDA for pediatric cardiomyopathy and it is the first drug to receive this designation since 1993. NP-6A4 increased cellular respiration and suppressed the doxorubicin-induced increase in reactive oxygen species (ROS) in hCAVSMSCs29. NP-6A4 also increased endothelial nitric oxide synthase (eNOS) and nitric oxide production in human coronary artery endothelial cells (hCAECs) [29]. Moreover, NP-6A4 is the only AT2R agonist that activates a positive feedback loop that increases AT2R expression in hCAVSMCs and hCAECs and human umbilical vein endothelial cells (hUVECs). These cellular effects of NP-6A4 were inhibited by AT2R-specific antagonist PD123319 or anti-AT2R siRNA [29]. Since NP-6A4 exerts protective effects on human cardiovascular cells that indicate its translational potential, we examined the effects of a four-week treatment with NP-6A4 on cardiac function, structure, microvascular rarefaction, and vascular damage in the heart tissues of obese female ZDF rats. We also analyzed the cardiac proteome to uncover new signaling mechanisms activated by NP-6A4-AT2R signaling in this obese female pre-clinical model with untreated obesity and pre-diabetes. Additionally, we confirmed that AT2R antagonist PD123319 would inhibit NP-6A4-AT2R signaling in obese female ZDF hearts. Here we report for the first time that NP-6A4-AT2R signaling activates protective cardiac autophagy in obese female ZDF rats and mitigates obesity-induced cardiac dysfunction and coronary microvascular damage.

## 2. Materials and Methods

A detailed Methods section is provided in the Appendix A. All animals were cared for in accordance with the Guidelines for the Care and Use of Laboratory Animals (National Institutes of Health publication 85–23). All animal procedures used in this study were approved prior to the beginning of these studies by the Animal Care and Use Committee (ACUC) of the University of Missouri-Columbia. The method of euthanasia is consistent with the American Veterinary Medical Association Guidelines for the Euthanasia of Animals. For euthanasia, rats were anesthetized with 3–4% isoflurane and euthanized by induction of double pneumothorax by opening the rib cage. The overall experimental protocol is outlined in Figure S1. Pilot experimental data on male ZDF rats that formed the basis for selecting the dose for NP-6A4 in this study are given in Figure S2. The mass spectrometry proteomics data have been deposited to the ProteomeXchange Consortium via the PRIDE partner repository with the dataset identifier PXD036596 [30].

All data are expressed as mean ± SEM and were analyzed with the use of GraphPad Prism 9.0. The Shapiro-Wilk test was used for assessing distribution normality. The 2-tailed Student *t*-test and 1-way analysis of variance (ANOVA) were used for data that were normally distributed. A value of *p* < 0.05 was considered to be statistically significant.

## 3. Results

### 3.1. Metabolic and Cardiac Phenotyping of 5-Month-Old ZDF-F Rats

**Figure 1:**
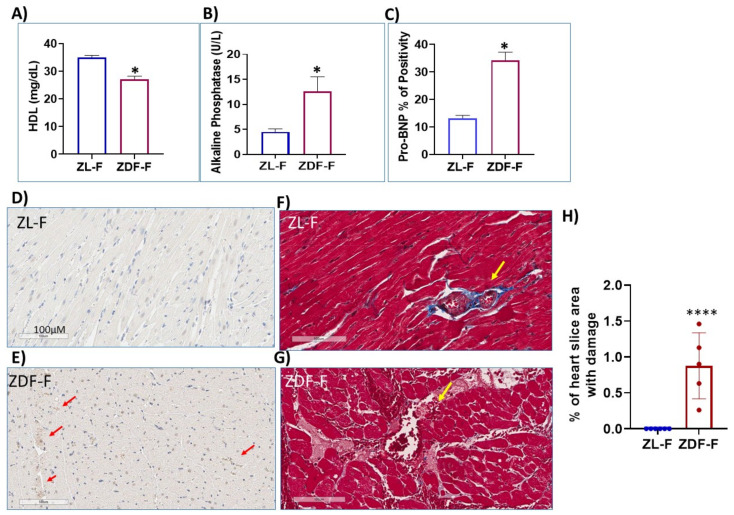
Obese female ZDF (ZDF-F) rats have reduced HDL but increased alkaline phosphatase in plasma and increased NT-pro-BNP (the heart failure marker) and microvascular damage in myocardium compared to healthy female ZL (ZL-F) rats. Five-month-old ZDF-F rats (n = 5) exhibit a reduction in plasma HDL (**A**) and an increase in alkaline phosphatase (**B**) and cardiac pro-BNP protein levels (**C**) compared to ZL-F rats (n = 6) * *p* < 0.05. (**D**,**E**) Representative images of cardiac sections of ZL-F and ZDF-F rats showing NT-pro-BNP expression using horseradish peroxidase staining. Red arrows mark brown clusters corresponding to NT-pro-BNP staining. (**F**,**G**) Representative images from Masson’s Trichrome staining of cardiac-tissue sections from ZDF-F rats. All ZDF-F rats had one or more vessels in the heart with pink-stained surrounding regions indicative of a vascular leak and edema and cell debris (blood vessel marked by yellow arrow) (**G**). None of the ZL-F rat hearts showed such damage (**F**). Scale bar: 100 µm. (**H**) shows the quantification of the damaged microvascular area as a percentage of the total whole heart section area for ZL-F and ZDF-F rats using Apeiro ImageScope annotation tools. **** *p* < 0.0001.

We reported previously that significantly higher levels of serum triglycerides were observed in young, adolescent, (5-month-old) obese ZDF-F rats fed with D12468 compared to age-matched healthy ZL-F rats [25]. Here we show that these ZDF-F rats also have reduced levels of plasma high-density lipoproteins (HDL) (Figure 1A) and increased levels of plasma alkaline phosphatase (ALP) (Figure 1B) compared to age-matched ZL-F rats. These observations are consistent with the metabolic effects of obesity and hyperglycemia on HDL and ALP in humans. It is well established that NT-proB-type Natriuretic Peptide (NT-pro-BNP) synthesized and secreted by the heart is increased during heart failure [31,32]. Immunohistochemistry using anti-NT-pro-BNP antibodies showed that compared to healthy ZL-F rat hearts (Figure 1C,D), ZDF-F rat hearts had increased brown color corresponding to anti-NT-Pro-BNP antibody positivity (Figure 1C,E; n = 5).

To further understand how obesity modulated coronary microvasculature in ZDF-F rat hearts, we examined trichrome-stained heart sections of 5-month-old ZDF-F and ZL-F rats. All ZDF-F rat hearts (n = 5), exhibited one or more blood vessels characterized by perivascular pink-stained areas similar to that shown in Figure 1G indicating a vascular leak, edema, and accumulation of dead cells. Such damage was not seen in ZL-F rat hearts (n = 6). The area of microvascular damage characterized by the pink staining was quantified using the Apeiro ImageScope annotation tools and presented as the percent of the total area of the whole heart section in Figure 1H. This coronary microvascular damage in 5-month-old ZDF-F rats is consistent with the high risk for coronary microvascular disease in young obese women.

### 3.2. Effects of NP-6A4 and NP-6A4 + PD123319 Treatments on Cardiac Functions of Obese and Hyperglycemic ZDF-F Rats

A hallmark of Grade III (reversible restrictive) heart failure is an E/A ratio (the ratio of peak blood flow velocity in the left ventricular during early diastolic relaxation (the E wave) to peak blood flow velocity in late diastole caused by atrial contraction (the A wave)) [33] of >2. Transthoracic echocardiography showed that at 17 weeks of age, obese and hyperglycemic ZDF-F rats treated with saline for 4 weeks (starting at 13 weeks of age) had an elevated E/A ratio (2.1 ± 0.048; lowest 1.98 and highest 2.22), one of the symptoms of reversible restrictive heart failure with preserved ejection fraction. However, ZDF-F rats treated with NP-6A4 for the same time had a significant reduction in their E/A ratio (E/A = 1.49 ± 0.14; Table 1; lowest: 1.03; highest 1.8). Both NP-6A4 and saline-treated ZDF-F rats had ejection fractions above 50% (Table 1); however, NP-6A4 treatment increased ejection fraction from 56.4 ± 2.1 to 63 ± 3. Additionally, saline-treated rats exhibited a prolonged period of isovolumic relaxation (22.3 vs. 18.6 ms) and isovolumic contraction times (13.6 vs. 9.8 ms) as well as an elevated myocardial performance index (0.44 vs. 0.39 ms), relative to rats treated with NP-6A4 (Table 1). Thus, NP-6A4 ameliorates cardiac diastolic and systolic impairments. NP-6A4 treatment also improved cardiac output, stroke volume, and myocardial radial strain and strain rate (Table 1) indicating that the baseline systolic impairments in 17-week-old ZDF-F rats were prevented by NP-6A4 treatment. To further confirm that NP-6A4 was acting through AT2R, we subjected a second cohort of ZDF-F rats to co-administration of NP-6A4 and the AT2R-specific antagonist PD123319. Co-administration of PD123319 with NP-6A4 inhibited all of the positive effects of NP-6A4 on cardiac functions of ZDF-F rats (Table 1) indicating that the salutary effects of NP-6A4 were mediated by AT2R activation. Neither NP-6A4 nor PD123319 significantly changed body weight, fasting plasma glucose and insulin levels, blood pressure, food and water intake, or urine output (Figure S3). Thus, the diastolic and systolic functional improvements in response to NP-6A4 treatment occurred despite the metabolic toxicity arising from obesity and insulin resistance in young ZDF-F rats. Conversely, a similar NP-6A4 treatment (10 mg/kg/day delivered by subcutaneous injection for 4 weeks) did not change any of the cardiac functional parameters of age-matched healthy female rats (Table S1).

### 3.3. Effects of NP-6A4 and NP-6A4 + PD123319 Treatments on Cardiac Capillary Density and Coronary Microvascular Damage in ZDF-F Rats

We reported previously that compared to ZL-F rats, ZDF-F rats exhibited cardiac microvascular rarefaction [25]. As shown in Figure 1G, young ZDF-F rat hearts also presented with visible microvascular damage characterized by pink staining indicative of an edema and/or vessel leak on one or more blood vessels. To determine if NP-6A4 mitigated these cardiac pathologies, we compared cardiac capillary density and cardiomyocyte size of ZDF-F rats treated with saline or NP-6A4. Staining the heart sections using *Griffonia simplicifolia* isolectin B4 (IB4) conjugated to Alexa Fluor 594 that detects endothelial cells showed that NP-6A4 treatment doubled the positivity of endothelial cell staining. 

**Figure 2:**
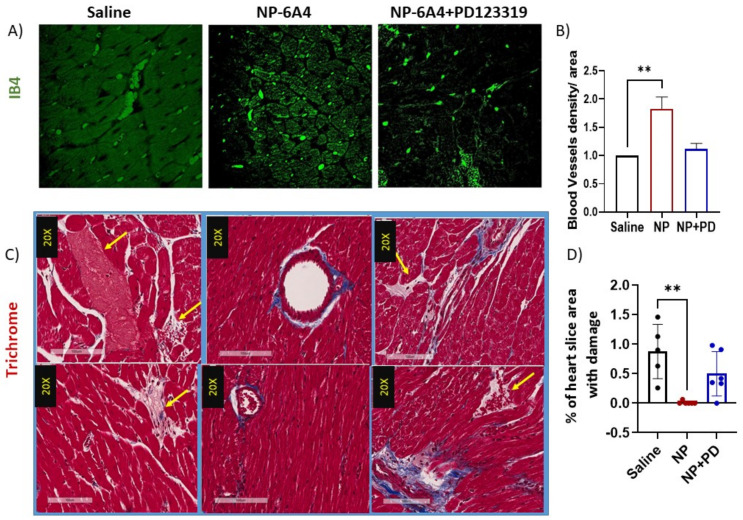
Effects of NP-6A4 and co-treatment with PD123319 on cardiac microvascular density and blood vessel damage in ZDF-F rats. Representative images of the heart sections of ZDF-F rats treated with saline and NP-6A4 and stained with endothelial marker Isolectin B4 (IB4) or Masson’s Trichrome are shown. NP-6A4-treated heart sections exhibit a doubling of IB4 signal indicating microvascular density increased by 200% in response to NP-6A4 compared to saline treatment (**A**,**B**). Co-treatment with PD123319 suppressed this effect (**A**,**B**) Scale bar: 50 µm. Trichrome-stained heart sections of ZDF-F rats treated with saline exhibit one or more regions of vascular damage indicated by pink-stained areas and marked by yellow arrows caused by cardiomyocyte loss and/or edema (**C**). This type of damage was not detected in five out of six ZDF-F rats treated with NP-6A4; however, ZDF-F rats treated with NP-6A4 + PD123319 showed similar vascular damage surrounding at least one blood vessel (**C**) Scale bar: 100 µm. (**D**) shows the quantification of damaged microvascular area as a percentage of the total whole heart slice area for ZDF-F rats treated with saline, or NP-6A4 or NP-6A4 + PD123319 (NP + PD) using Apeiro ImageScope annotation tools. ** *p* < 0.01.

Thus, cardiac microvascular density was doubled in NP-6A4-treated rats (Figure 2A,B). Co-treatment with PD123319 attenuated this increase in cardiac microvascular density (Figure 2A,B). Therefore, NP-6A4 acts through AT2R to increase cardiac microvascular density in young, obese, and hyperglycemic ZDF-F rats. We also analyzed trichrome-stained heart sections from rats in all treatment groups to assess if treatment with NP-6A4 or co-treatment PD123319 modulated cardiac microvascular damage. All saline-treated ZDF-F rat hearts (n = 5) showed one or more blood vessels with the type of perivascular damage shown in Figure 1G and Figure 2C. However, 5 out of 6 NP-6A4-treated rat hearts did not have blood vessels with similar microvascular damage (−625% reduction) (Figure 2C). Conversely, all rats treated with NP-6A4 and PD123319 (co-treatments) had one or more blood vessels with microvascular damage (Figure 2C) in their heart sections. The area of microvascular damage characterized by the pink staining was quantified in NP-6A4-treated and NP-6A4 + PD123319-treated (NP + PD) ZDF-F rats using the Apeiro ImageScope annotation tools and presented as the percentage of the total area of the whole heart section in Figure 2D. Thus, the beneficial effects of NP-6A4 on this type of microvascular damage were abrogated by PD123319 co-treatment.

### 3.4. Effects of NP-6A4 and NP-6A4 + PD123319 Treatments on Cardiomyocyte Hypertrophy, Fibrosis and Cardiac AT2R Expression in ZDF-F Rats

**Figure 3:**
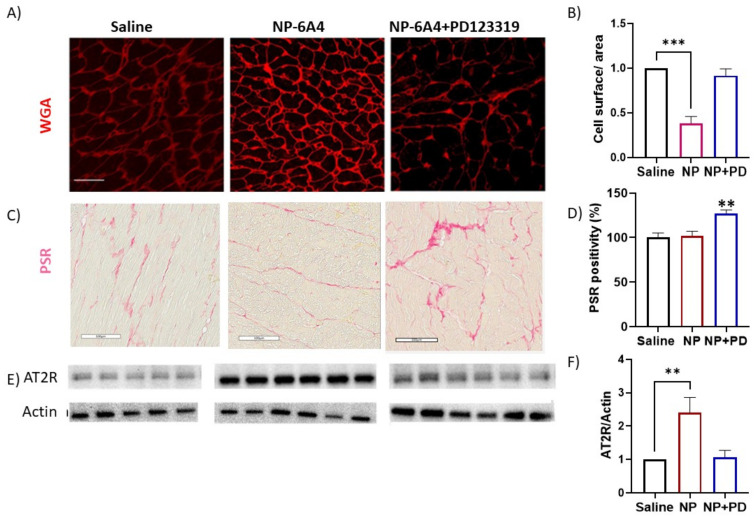
Effects of NP-6A4 and co-treatment with PD123319 on cardiomyocyte hypertrophy, cardiac fibrosis, and AT2R protein expression in ZDF-F rats. Representative images of heart sections of ZDF-F rats treated with wheat germ agglutinin (WGA) staining and picrosirius staining are shown. WGA staining (**A**,**B**) show that rats treated with NP-6A4 have a 61.7% reduction in cardiomyocyte surface area compared to rats treated with saline or NP-6A4 + PD123319 (NP+PD). Scale bar: 50 µm. Quantification of picrosirius red (PSR) staining of collagen in the heart sections of ZDF-F rats showed that there was no difference in the PSR staining between saline and NP-6A4 treated rats, but NP6A4 + PD123319 (NP+PD) treatment that inhibited all AT2R signaling increased PSR positivity by 27% (**C**,**D**). Scale bar: 100 µm. Immunoblotting of heart tissue lysates from ZDF-F rats with anti-AT2R antibody (**E**) showed that AT2R expression was increased by 240% in NP-6A4- compared to saline- or NP-6A4 + PD123319-treated rats (**F**). ** *p* < 0.01, *** *p* < 0.001.

We reported previously that NP-6A4 suppressed cardiomyocyte hypertrophy in male Zucker Obese rats [28]. Staining with wheat germ agglutinin (WGA) conjugated with Alexa Fluor 647 to detect the cardiomyocyte cell membrane showed that NP-6A4 treatment reduced cardiomyocyte area (−263%) in ZDF-F rats, and this effect was attenuated by co-treatment with PD123319 (Figure 3A,B). Thus, NP-6A4 acts through AT2R to reduce cardiomyocyte hypertrophy. We also noticed that rat heart sections stained with WGA from both saline-treated and NP-6A4 + PD123319-treated rats had a diffused appearance of cardiomyocyte membranes while those treated with NP-6A4 had a more defined appearance (Figure 3A).

We reported previously that young ZDF-F rats did not show an increase in fibrosis compared to healthy ZL-F rats [25]. Picrosirius red (PSR) staining to detect collagen levels in the heart sections showed that NP-6A4 treatment did not change the extent of PSR staining in ZDF-F rats (Figure 3C,D). This is not surprising since ZDF-F rats at this age actually did not exhibit cardiac fibrosis [25] and NP-6A4 treatment did not change baseline collagen levels. However, co-treatment with PD123319 that inhibits AT2R significantly increased PSR staining (27%) in ZDF-F rat hearts (Figure 3C,D). This observation suggests that inhibition of AT2R (by PD123319) increases cardiac fibrosis in obese and hyperglycemic ZDF-F rat.

We reported previously that 5-month-old ZDF-F rats exhibited significant suppression (−238%) of AT2R expression in the heart compared to age-matched healthy ZL-F rats [25]. To determine if treatment with NP-6A4 or co-treatment with PD123319 modulated AT2R expression in ZDF-F rat hearts, we performed an immunoblotting analysis of the heart lysates of ZDF-F rats from all treatment groups using an anti-AT2R antibody.

NP-6A4 treatment increased AT2R protein expression in ZDF-F rat hearts by 240% (Figure 3E,F), but this increase in AT2R protein levels was attenuated by co-treatment with PD123319. These data are consistent with our observation that NP-6A4 could increase AT2R expression in human cardiovascular cells and male Zucker Obese rat hearts, and inhibition of AT2R, either by PD123319 or anti-AT2R siRNA, attenuated the NP-6A4-induced increase in AT2R in human cells [28,29]. Moreover, PD123319-mediated suppression of AT2R could have contributed to increased cardiac fibrosis in ZDF-F rats treated with NP-6A4 + PD123319.

### 3.5. Cardiac Proteome Analysis Shows Activation of Superpathway of Inositol Compounds by NP-6A4-AT2R Signaling in ZDF-F Rat Hearts

**Figure 4:**
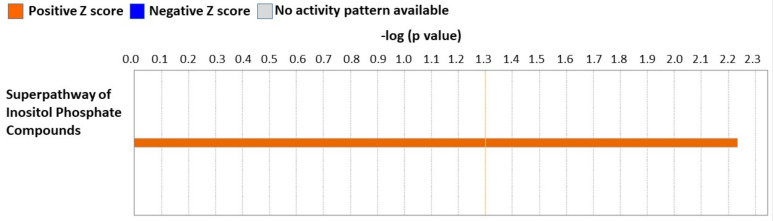
IPA-predicted activation of the canonical pathway Superpathway of Inositol Compounds in the heart tissues of ZDF-F ats treated with NP-6A4IPA-predicted Z score: 2.234. The orange color indicates the positive Z score and activation of the pathway.

To determine how NP-6A4 acting through AT2R mechanistically modulates cardiac damage caused by obesity and hyperglycemia, we compared the cardiac proteome of ZDF-F rats treated with saline, NP-6A4, or NP-6A4 + PD123319, as detailed in Appendix A. This analysis resulted in the identification of a cardiac proteome consisting of 4500 proteins. The global sum normalization method followed by a two-sample unequal variance *t*-test showed 112 proteins differentially expressed between saline- and NP-6A4-treated ZDF-F rats, and 299 proteins differentially expressed between NP-6A4- and NP-6A4 + PD123319 treated ZDF-F rats (*p* < 0.05). A list of these differentially expressed proteins is given in Tables S2 and S3. Analysis of these differentially expressed proteins between NP-6A4- versus saline-treated groups using ingenuity pathway analysis (Figure 4) identified that the ‘Superpathway of Inositol Phosphate Compounds’ canonical pathway is activated in response to NP-6A4 treatment (IPA-predicted value: Z score = 2.234) in ZDF-F rats. This pathway is critical for cardiac health because it is down-regulated in pre-clinical models for hypertrophic cardiomyopathy [34].

### 3.6. NP-6A4 Induced Cardiac Autophagy Activation in Obese ZDF-F Rats and PD123319 Suppressed This Effect

**Figure 5:**
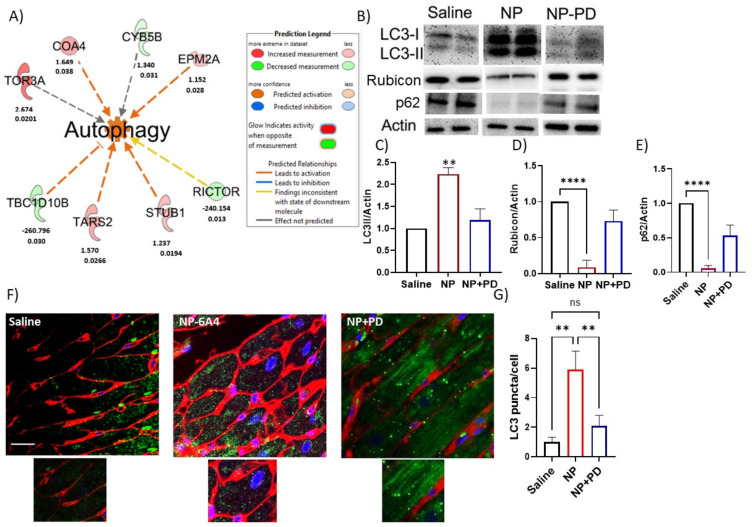
Effect of treatment with NP-6A4 or co-treatment with PD123319 on the cardiac autophagy status of ZDF-F rats. (**A**) New autophagy signaling pathway consisting of 8 proteins activated in response to NP-6A4 in ZDF-F rat heart as predicted by Ingenuity Pathway Analysis of the differentially expressed proteins between saline and NP-6A4 treated rat hearts (orange color indicates activation: see Prediction legend) in the Diseases and Functions category. The fold change of each protein in NP-6A4-treated rat hearts compared to those treated with saline (top number below each protein) and *p* value (bottom number below each protein) are shown. Z score = 1.664; *p* value = 0.005. (**B**) Representative images of immunoblotting analysis of cardiac lysates from ZDF-F rats treated with saline, NP-6A4, or NP-6A4 + PD123319 and probed with anti-LC3, anti-Rubicon, anti-p62, and anti-Actin antibodies are shown. An increase in LC3-I and LC3-II levels and a reduction in Rubicon and p62 levels is seen in ZDF-F rat heart lysates treated with NP-6A4 compared to those treated with saline or NP-6A4 + PD123319. Graphs show quantification of LC3-II (**C**), Rubicon (**D**), and p62 (**E**) in ZDF-F rat heart lysates after normalization to Actin. NP-6A4 significantly increased LC3-II, but suppressed Rubicon and p62. (**F**) Representative images of immunohistochemistry of cardiac tissues from ZDF-F rats treated with saline, NP-6A4, or NP-6A4 + PD123319 and immunostained with (1) anti-LC3 antibodies (green) to visualize autophagic vesicles, (2) wheat germ agglutinin (red) to define boundaries of cardiomyocytes and (3) DAPI (blue) to visualize the nucleus. Zoomed-in highlights are shown in the row below. The scale bar (30 µm) is marked as a white line. Cardiomyocyte boundaries are well defined in the heart tissues of NP-6A4-treated rats compared to those treated with saline, but co-treatment with PD123319 made cardiomyocyte boundaries even more diffused. (**G**) Autophagic dot numbers significantly increased in rat hearts treated with NP-6A4, and this was attenuated by co-treatment with PD123319. The increase in LC3-II seen in panel C correlates with the increase in autophagosome puncta seen in E and F indicating induction of autophagy in NP-6A4-treated ZDF-F rat hearts. ** *p* < 0.01; **** *p* < 0.0001; ns: not significant.

IPA analysis of the predicted ‘Diseases and Functions’ showed that a new 8-protein autophagy pathway is activated in NP-6A4-treated ZDF-F rat hearts (Figure 5A). Two key proteins involved in this pathway are Rictor, a critical component of the mammalian target for rapamycin (mTOR) Complex 2 and TBC1 Domain Family Member 10B (TBC1D10B) that are down-regulated by over 200 folds in NP-6A4-treated ZDF-F rat hearts. Rictor expression is increased in conditions where autophagy and mitophagy are inhibited [35,36].

TBC1D10B is a Rab GTPase activating protein (Rab GAP) that interacts with the autophagy marker ATG8/LC3 [37] and is involved in exocytosis [37,38]. To date, there are no reports that show an autophagy activation pathway that connects AT2R, Rictor and TBC1D10B. Since our IPA-predicted autophagy pathway had a Z score of only 1.664, we performed additional studies to validate the IPA-predicted activation of autophagy by NP-6A4-AT2R signaling in ZDF-F rat hearts. Microtubule-associated protein 1A/1B-light chain 3 (LC3) protein is ubiquitously distributed in mammalian cells. During autophagy, the cytosolic form of LC3 (LC3-I) is engulfed and becomes conjugated to phosphatidylethanolamine to form LC3-phosphatidylethanolamine conjugate (LC3-II), which is recruited to autophagosomal membranes [39,40,41]. As the autophagosome fuses with lysosome to form autolysosome and degradation of intra-autophagosomal proteins occur, LC3-II is degraded [39,40,41]. Therefore, the detection of LC3-II is a reliable method to validate the activation of autophagy. Immunoblotting of ZDF-F rat heart lysates using anti-LC3 antibodies showed that NP-6A4-treated ZDF-F rat hearts had a substantial increase in LC3-II levels compared to saline-treated groups and this effect was attenuated by co-treatment with PD123319 (Figure 5B,C).

Rubicon is a Beclin 1–binding protein that suppresses autophagy and endocytosis, and an increase in Rubicon protein levels is associated with impairment of autophagy [42,43,44]. Therefore, to further confirm the status of autophagy in the heart tissues of ZDF-F rats treated with NP-6A4 or co-treated with NP-6A4 and PD123319, we evaluated the expression levels of Rubicon protein in the heart tissue lysates of these rats via immunoblotting with an anti-Rubicon antibody. This analysis showed that Rubicon protein expression was significantly suppressed by treatment with NP-6A4 in ZDF-F rat hearts, but this suppression was reversed by PD123319 (Figure 5B,D).

To further confirm that NP-6A4 activates autophagy, we checked expression levels of the autophagy receptor, the p62 protein. The p62 protein, also known as the protein Sequestosome1 (p62/SQSTM1), is a classical selective autophagy receptor that serves as a link between autophagy and ubiquitin–proteasome system, cell metabolism, and apoptosis [45]. Activation of autophagy coincides with the degradation of p62 [46]. Consistent with the autophagy activation by NP-6A4, the heart tissue lysates of NP-6A4-treated ZDF-F rats had a significant reduction of p62 protein levels compared to those treated with saline (Figure 5B,E). Moreover, PD123319-co-treatment prevented NP-6A4-induced reduction in p62 protein (Figure 5B,E). To identify differences in the distribution of LC3-II puncta in ZDF-F rat hearts in response to NP-6A4 or co-treatment with NP-6A4 and PD123319, we performed immunohistochemistry of heart sections from all treatment groups using an anti-LC3 antibody. The number of autophagic dots increased with NP-6A4 treatment and PD123319 co-treatment suppressed this effect (Figure 5F,G). Saline-treated ZDF-F rat hearts exhibited a reduction in the definition of cardiomyocyte membrane compared to NP-6A4-treated rats (Figure 5F,G) suggesting a loss of membrane integrity in the saline-treated ZDF-F rat hearts. Co-treatment with PD123319 worsened the definition of cardiomyocyte membrane further ((Figure 5E,F). This indicates that inhibition of AT2R signaling caused by co-treatment with PD123319 worsens cardiomyocyte membrane integrity.

### 3.7. Inhibition of AT2R by Co-Treatment with PD123319 Induced Inflammatory Reelin Signaling Pathway in the Heart

To get further mechanistic insight into the inhibition of NP-6A4-AT2R signaling by PD123319, we performed an IPA analysis of differentially expressed proteins identified from the cardiac proteome analysis of ZDF-F rats that were treated with NP-6A4 versus those treated with NP-6A4 + PD123319.

**Figure 6:**
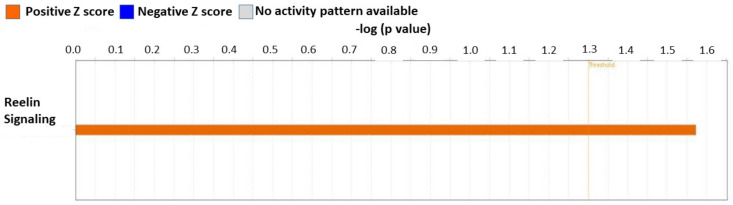
IPA-predicted activation of Reelin Signaling in the heart tissues of ZDF-F rats co-treated with AT2R antagonist PD123319. IPA-predicted Z score: 2.0. Orange color indicates activation of the pathway.

IPA analysis to identify canonical pathways modulated by PD123319 co-treatment showed that the Reelin signaling canonical pathway was activated (Z score = 2.0, *p* < 0.0001) in the heart tissues of ZDF-F rats treated with NP-6A4 + PD123319 (Figure 6). Reelin signaling is implicated in increased endothelial-leukocyte adhesion and macrophage accumulation in lesions and the development of atherosclerosis [47,48]. Therefore, activation of the Reelin pathway in the hearts of ZDF-F rats in response to PD123319 co-treatment could have a role in the increased coronary vascular damage observed in ZDF-F rats treated with NP-6A4 + PD12339 in Figure 2C. To our knowledge, this is the first report that indicates AT2R regulates Reelin signaling.

### 3.8. Inhibition of NP-6A4-AT2R Signaling in ZDF-F Rat Heart by Co-Treatment with PD123319 Induces Signaling Networks That Decrease ATP Concentration, and Increase Muscle Cell Death

IPA-predicted ‘Diseases and Functions’ analysis of the above set of differentially expressed proteins in the heart tissues of ZDF-F rats subjected to treatments with NP-6A4 versus NP-6A4 + PD123319 identified two signaling networks that are induced by PD123319 co-treatment that may be involved in the detrimental effects of AT2R inhibition by PD123319. 

**Figure 7:**
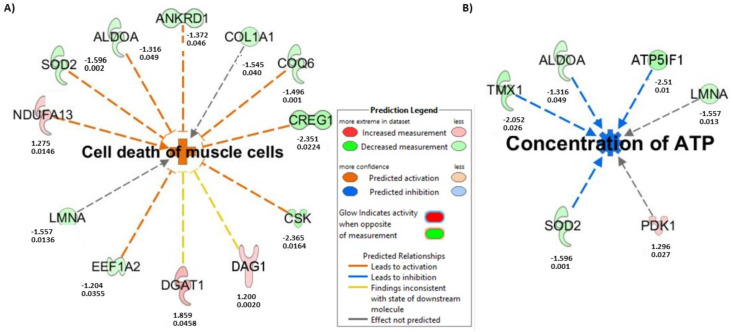
Effects of co-treatment with AT2R antagonist PD123319 on cardiac signaling of young ZDF-F rats. IPA analysis of differentially expressed proteins between NP-6A4 and NP-6A4+ PD123319-treated rat hearts 6 predicted that co-treatment with PD123319 resulted in activating a signaling network that (**A**) increases cell death of muscle cells (orange indicates activation: Z score: 1.943; *p* value = 0.0015) and another signaling network that (**B**) suppresses ATP concentration (blue indicates suppression: Z score: −1.982; *p* value =0.008) in the Diseases and Functions Category. Fold changes of each protein (top number below each protein) in heart lysates of ZDF rats treated with NP-6A4 + PD123319 versus those treated with NP-6A4 alone and corresponding *p* values (bottom number below each protein) are shown in (**A**,**B**).

The first signaling network is predicted to increase muscle cell death (Figure 7A, Z score = 1.943). This is consistent with increased suppression of autophagy in the heart tissues of ZDF-F rats treated with NP-6A4 + PD123319 compared to those treated with only NP-6A4. The second signaling network is predicted to suppress ATP concentration (Figure 7B, Z score = −1.982). Since ATP demand is higher in cells undergoing autophagy, inducing a signaling network that suppresses ATP concentration is consistent with the suppression of autophagy in the heart tissues of ZDF-F rats subjected to co-treatment with NP-6A4 and PD123319.

Additional studies are warranted to confirm that the cardiovascular detrimental effects of AT2R inhibition involve IPA-predicted networks that contribute to muscle cell death and reduce ATP levels in the cells.

## 4. Discussion

Multiple clinical reports and reviews have repeatedly shown that ischemic heart disease and impaired coronary microvascular function are prevalent in obese and diabetic patients [1,2,3,4,5,6,7,8,9,10,11,12]. In the U.S., more than 80% of patients who have heart failure with preserved ejection fraction are obese and/or diabetic [49]. Obese and diabetic patients with coronary microvascular dysfunction and left ventricular dysfunction with preserved ejection fraction are shown to be at higher risk for hospitalization and heart failure. Clinical trials with anti-hypertensive drugs have reported neutral effects in this population of patients [50]. Obese and diabetic women, particularly those of childbearing age, have additional risk factors than age-matched obese men due to pregnancy-associated cardiovascular complications that cause increased cardiovascular risk during pregnancy and after childbirth [50]. Although weight loss and exercise improve cardiac function and heart failure in these obese patients, their reduced exercise capacity and social conditions often prevent the successful use of these potential solutions. Thus, a pharmaceutical intervention that can improve coronary microcirculation and left ventricular dysfunction with preserved ejection fraction in obese and diabetic patients even in the presence of metabolic toxicity caused by obesity and diabetes (chronic inflammation, hyperglycemia, hyperlipidemia, hypercholesteremia, increased body weight, and adiposity) will be very useful in protecting their heart and vasculature while leading them through a regimen of exercise and weight loss to improve their health.

The AT2R, encoded by the X-linked *Agtr2* gene, exhibits sex differences in cardiovascular and renal expression [25,51,52,53]. A combination of obesity and hyperglycemia suppresses cardiac AT2R in females [25]. Activating AT2R signaling by agonists [27,28,29,54,55] and increasing the *Agtr2* gene copy number by genetic manipulation in murine models improve cardiac repair and enhance cardiac function [56,57,58]. Clinically, loss of AT2R expression due to the intronic G1675A or A1818T polymorphism in men is associated with impaired pulse pressure, increased arterial stiffness, and kidney dysfunction [59,60]. Compound 21 (C21) is the only AT2R agonist used in clinical trials for idiopathic pulmonary fibrosis and COVID-19 [61,62], but not for heart disease, and there are no reports that show C21 increases *Agtr2* gene expression. Thus, there are no AT2R agonists currently used in the clinic to treat cardiovascular diseases by increasing AT2R gene expression and signaling.

NP-6A4 is an AT2R agonist that could increase the expression and signaling of AT2R in human cardiovascular cells and obese male rat hearts as we have reported before [28,29]. Data presented here confirms that NP-6A4 treatment increased AT2R expression via a feed-forward loop that requires AT2R signaling in obese female rat hearts. Moreover, inhibition of the AT2R function by PD123319 attenuated the NP-6A4-mediated increase in both AT2R expression and signaling. Four-week NP-6A4 treatment effectively mitigated several parameters of obesity- and hyperglycemia-induced cardiac diastolic and systolic dysfunction with preserved ejection fraction despite the fact that these female rats (ZDF-F) continued to have elevated body weight and hyperglycemia. Their blood pressure levels were unaffected by this systemic NP-6A4 treatment and, therefore, the beneficial effects of NP-6A4 were not mediated via blood pressure reduction in ZDF-F rats. Moreover, cardiomyocyte hypertrophy induced by obesity and hyperglycemia in ZDF-F rats [25] was attenuated by NP-6A4 treatment. These data are consistent with the effects of NP-6A4 treatment on young male obese (Zucker Obese) rats who also suffer from metabolic toxicity induced by obesity and hyperglycemia and cardiac dysfunction with preserved ejection fraction as we have reported [28]. All of these beneficial effects of NP-6A4 were attenuated by co-treatment with AT2R antagonist PD123319. Thus NP-6A4’s cardioprotective effects require functional AT2R.

Data presented here highlight two important effects of NP-6A4-induced AT2R activation on coronary microvascular damage in an obese and hyperglycemic female rat model. First, NP-6A4 acting through AT2R increased cardiac capillary density despite metabolic diseases. Second, coronary vessel damage (indicated by pink staining around the blood vessel in trichrome-stained cardiac sections shown in Figure 1G and Figure 2C) was absent in five out of six NP-6A4-treated ZDF-F rat hearts examined. Thus, NP-6A4 treatment seems to attenuate coronary microvascular damage in this obese and hyperglycemic female pre-clinical model. Conversely, inhibition of AT2R by co-treatment with PD123319 nullified both these effects. This is consistent with our previous observation that NP-6A4 activates human coronary endothelial cell functions and increases endothelial nitric oxide synthase expression and activity [29].

We showed previously that obese and pre-diabetic male rats that were treated for two weeks using a lower dose of NP-6A4 (1.8 mg/kg/day) exhibited improvement in cardiac functions and structure [28]. In these male rats, the two-week low-dose NP-6A4 treatment suppressed cardiomyocyte hypertrophy (−121.9%) and increased cardiac capillary density (125%) and AT2R protein expression (135%). The current study shows that (1) obese and pre-diabetic female rats also benefit from NP-6A4 treatment, and (2) they actually responded better to the four-week, high dose NP-6A4 treatment since their cardiomyocyte hypertrophy was suppressed by −263%, cardiac capillary density increased by 200%, and cardiac AT2R protein expression increased by 240%. Thus, the current study shows that a high dose of NP-6A4 treatment for a longer period is more effective in improving cardiac function and structure in obese and pre-diabetic rats and does not seem to cause any negative side effects (well-tolerated). Neither the low-dose NP-6A4 treatment nor the high-dose NP-6A4 treatment mitigated obesity or hyperglycemia in males and females. Thus, NP-6A4 could protect cardiomyocytes (as evidenced by the reduction in cardiomyocyte hypertrophy) and vascular and endothelial cells (as evidenced by reduced microvascular damage and increased capillary density) and thus improve cardiac function in conditions of untreated obesity and hyperglycemia.

Our preliminary cardiac proteome analysis followed by IPA analysis of differentially expressed proteins resulted in a surprising discovery that NP-6A4 activated a new autophagy pathway in the heart tissues of obese and hyperglycemic ZDF-F. Since previous studies on neonatal cardiomyocytes have shown that AT2R antagonizes autophagy [63,64], we performed additional experiments to verify that NP-6A4 actually activated autophagy machinery through AT2R in ZDF-F rat hearts. Our observation that NP-6A4 increased autophagy marker LC3-II and suppressed both autophagy receptor p62 and autophagy inhibitor Rubicon in ZDF-F rat hearts further confirmed that NP-6A4-AT2R signaling activated autophagy in ZDF-F rat hearts. Since this autophagy activation coincided with improved cardiac function and reduced cardiac structural damage (a reduction in cardiomyocyte hypertrophy and coronary vascular damage and an increase in capillary density) in ZDF-F rats, it is reasonable to conclude that NP-6A4-AT2R-induced autophagy is cardioprotective in conditions of obesity and hyperglycemia. The cardioprotective role of NP-6A4-AT2R-induced autophagy is further confirmed by the fact that inhibition of AT2R by PD123319 co-treatment attenuated autophagy as well as worsened cardiac functions and cardiac structural damage in ZDF-F.

Our observation that NP-6A4, an FDA-designated cardiomyopathy drug, could activate cardioprotective autophagy and attenuate cardiac dysfunction and coronary microvascular damage in conditions of untreated obesity and hyperglycemia in our female preclinical model has high clinical significance. A safe and effective treatment to restore impaired cardioprotective autophagy to mitigate heart disease with preserved ejection fraction in obese and diabetic patients is currently an unmet medical need. Since NP-6A4 activates human coronary endothelial cell functions and mitigates coronary microvascular damage, and attenuates cardiac dysfunction with preserved ejection fraction, NP-6A4 is a safe autophagy inducer to protect female heart subjected to metabolic toxicity caused by obesity and hyperglycemia. The NP-6A4-induced new autophagy pathway predicted by IPA involves 8 proteins. The >200 fold suppression of mTORC2 component Rictor and TBC1D10B by NP-6A4 is particularly noteworthy. There are no previous reports that indicate the expression of Rictor and TBC1D10B are co-regulated by AT2R and contribute to AT2R-induced autophagy in the heart. Thus, additional studies are warranted to fully understand how NP-6A4-AT2R signaling suppresses Rictor and TBC1D10B and the role of these molecules in AT2R-induced cardioprotective autophagy in obesity and hyperglycemia.

Data presented here also highlights the role of AT2R in the female heart in the setting of chronic inflammation arising from obesity and hyperglycemia. IPA analysis of differentially expressed proteins in the cardiac proteome of ZDF-F rats treated with NP-6A4 versus saline indicate that NP-6A4 induces activation of the superpathway of inositol phosphate compounds in ZDF-F rat hearts. Conversely, inhibition of AT2R by co-treatment with PD123319 suppresses an IPA-predicted pathway that regulates ATP concentration in ZDF-F rat hearts. Taken together, these data suggest that AT2R has a crucial role in regulating cardiac energy metabolism. Moreover, inhibition of AT2R by PD123319 co-treatment activated two IPA-predicted signaling pathways that contribute to cell death (Cell death of muscle cells pathway: Figure 7A) and vascular damage (Reelin signaling pathway that promotes atherosclerosis). Collectively these data suggest that activation of AT2R expression and signaling is critical for the cardiac health of obese female rats and inhibition of AT2R exacerbates cardiac and vascular pathology.

Limitations of this study: Our preliminary cardiac proteome analysis of ZDF-F rats treated with saline, NP-6A4, and NP-6A4 + PD123319 (NP + PD) did not show significant differences between cardiac proteomes of different experimental groups as per the Benjamini-Hochberg method. It is conceivable that since all rats belong to the same strain (ZDF-F) and had metabolic disease (obesity and pre-diabetes) and these conditions were not changed by any of the treatments, the differences only in a small subset of cardiac proteins could have been sufficient to drive the protective effects of treatment with AT2R agonist NP-6A4, or inhibition of these protective effects by co-treatment with AT2R antagonist PD123319. We recognize that a larger sample size is required to achieve significant differences between the entire cardiac proteome of these different experimental groups. Thus, additional experiments are warranted to achieve statistical significance between the cardiac protein profiles of different experimental groups. However, it is noteworthy that our IPA analysis using the subset of proteins that had a *p* value ≤ 0.05 identified from our pairwise analysis using Student’s *t* test of the proteome data from NP-6A4-treated rats to saline-treated rats or NP-6A4-treated to NP + PD-treated rats generated useful information. Results of the IPA analysis indicated that cardiac autophagy is activated by NP-6A4 treatment in ZDF-F rats. This information is new since previous studies suggested that AT2R inhibited autophagy [58,59]. Our additional experiments further confirmed this hypothesis as shown in Figure 5. The IPA analysis of this subset of proteins also identified new canonical pathways (Figure 4 and Figure 6) and new networks (Figure 7) associated with AT2R that we have reported here. Additional experiments are warranted to further confirm AT2R signaling modulates these pathways and networks.

## 5. Conclusions

In summary, the present study shows that NP-6A4 acting through AT2R improves cardiac functions and mitigates both cardiomyocyte structural damage and coronary microvascular damage in obese and hyperglycemic female rats that mimic the metabolic and cardiac pathology of young obese and hyperglycemic women. Mechanistically, NP-6A4 induced new AT2R-mediated autophagy in the heart tissues of these rats to protect the heart in the presence of untreated obesity and hyperglycemia. Conversely, inhibition of AT2R signaling by co-treatment with PD123319 attenuated NP-6A4-mediated improvements in cardiac functions and structure and cardiac autophagy.

Impairment of autophagy induced by obesity and hyperglycemia is a pivotal cause of heart failure, and currently, there are no effective drugs that can induce cardiac autophagy safely in patients with obesity and/or hyperglycemia. We show for the first time that NP-6A4-AT2R signaling activates whereas AT2R inhibition prevents protective autophagy in obese and hyperglycemic female hearts. These data suggest that NP-6A4 can be a useful cardiovascular protective drug that activates protective autophagy and mitigates cardiac damage in highly vulnerable obese females with elevated risk for cardiovascular death.

## Figures and Tables

**Table 1:** Cardiac functional parameters of ZDF-F rats that were modulated by treatment with NP-6A4 alone or co-treatment with PD123319. Statistical significance is marked by *p* values in bold.

Cardiac Parameters	Saline (N = 5)	NP-6A4 (N = 6)	NP-6A4 + PD123319 (N = 6)	Saline vs. NP-6A4 (*p* Value)	NP-6A4 vs. NP-6A4 + PD123319 (*p* Value)
Heart rate	247 ± 9	310 ± 23	269 ± 10	**0.034**	0.15
E/A	2.11 ± 0.048	1.49 ± 0.14	1.84 ± 0.09	**0.006**	**0.08**
Stroke Volume (SV) (µL)	185 ± 9	253 ± 31	165 ± 20	**0.034**	**0.035**
Ejection Fraction (EF)	56.4 ± 2.1	63 ± 3	56.3 ± 3.2	0.059	0.167
Cardiac Output (CO)	50 ± 3	80 ± 10	47 ± 5	**0.012**	**0.011**
Radial Strain (Pk%)	22.8 ± 2.77	38.0 ± 5.28	20.0 ± 3.67	**0.019**	**0.018**
Radial Strain Rate (Pk1/s)	3.69 ± 0.43	6.44 ± 1.15	3.55 ± 0.51	**0.033**	**0.037**
Isovolumic Relaxation time (IVRT (ms)	22.3 ± 0.617	18.6 ± 1.01	24.5 ± 0.935	**0.022**	**0.002**
Isovolumic Contraction time (IVCT) (ms)	13.6 ± 0.835	9.78 ± 0.45	14.1 ± 1.12	**0.004**	**0.009**
Systolic Time (Syst T) (ms)	117 ± 3.065	102 ± 5.33	120 ± 3.329	0.056	**0.016**
Myocardial Performance Index (MPI)	0.44 ± 0.027	0.39 ± 0.01	0.48 ± 0.02	0.06	**0.021**

## Data Availability

The original contributions presented in the study are included in the article.

## Supplementary Materials

The following supporting information can be downloaded at: https://www.mdpi.com/article/10.3390/cells12101373/s1, Figure S1: Experimental Strategy for drug treatments of female ZDF rats; Figure S2: Effects of different doses of NP-6A4 on some of the cardiac functions of male ZDF rats; Figure S3: Body weight, fasting plasma glucose and insulin, 24-h food and water consumption, 24-h urine output, and blood pressure of ZDF-F rats treated with saline, NP-6A4, or NP-6A4 + PD123319; Figure S4: PCA plot of treatment groups; Figure S5: Negative control for the immunohistochemistry using an anti-LC-3 antibody. Table S1: Effects of four-week NP-6A4 treatment on cardiac parameters of healthy female Wistar (Wistar-F) rats; Table S2: Differentially expressed proteins in ZDF-F rats treated with saline and NP-6A4; Table S3: Differentially expressed proteins in ZDF-F rats treated with NP-6A4 and NP-6A4 + PD123319.

## Appendix A

### Appendix A.1. Animals

All animal procedures used in this study were approved prior to the beginning of these studies by the University of Missouri Animal Care and Use Committee. All animals were cared for in accordance with the Guidelines for the Care and Use of Laboratory Animals (National Institutes of Health publication 85–23). Zucker diabetic fatty (ZDF) female (ZDF-F) rats were purchased from Charles River Laboratories and were fed with a high-fat diet (Research Diet 12,468 with 10 kcal% soy protein and 48 kcal% lard) to induce hyperglycemia starting at the age of seven weeks. Rats were given numbers after randomization based on body weight and before starting treatments and only the numbers were used for identifying the animals during procedures to ensure studies/analyses were performed in a blind manner. Figure S1 shows the experimental strategy for treatment with saline, NP-6A4 and NP-6A4 + PD123319.

### Appendix A.2. Drug Treatments

NP-6A4 (10 mg/kg/day) and PD123319 (5 mg/kg/day) were dissolved in bacteriostatic saline and delivered via subcutaneous injections for four weeks. The dose of NP-6A4 was determined from a pilot study on male ZDF rats (n = 14) receiving different doses (5 mg/kg/day [n = 5] and 10 mg/kg/day [n = 5]) of NP-6A4 dissolved in saline compared to those receiving vehicle (saline: [n = 4]) alone. The mitral E/e′ (MVE/e′) ratio is a noninvasive measure of left ventricular filling pressure. After four weeks of treatment, preliminary echocardiography analysis showed that the dose of 10 mg/kg/day was more effective in reducing the E/e’ ratio (the ratio of early diastolic mitral inflow velocity to early diastolic mitral annulus velocity) that indicates a reduction in left ventricular filling pressure in these rats (Figure S2A). NP-6A4 (10 mg/kg/day) was also more effective in reducing the left ventricular anterior wall thickness in systole (LVAW(s)) (Figure S2B). Based on this data, a dose of 10 mg/kg/day was selected for the present study.

### Appendix A.3. Body Weight, Fasting Plasma Profile and Blood Pressure

Body weight and fasting plasma glucose and insulin levels of ZDF-F rats at different stages of study as determined by the procedures described previously for obese and diabetic rat models [25,65,66] and mean ± SEM is shown in Figure S3A–C. Measurement of 24-h food and water intake was repeated on three different days and means ± SEM are shown in Figure S3D. Blood pressure was measured non-invasively on conscious rats using a CODA volume pressure recording tail-cuff system (Kent Scientific Corporation, Torrington, CT, USA) as described previously [67] and means ± SEM are shown in Figure S3E. Drug treatments did not change any of these parameters significantly.

### Appendix A.4. Echocardiography

Transthoracic echocardiography was performed under inhaled isoflurane anesthesia (1.5% maintenance) utilizing a Vevo2100 rodent ultrasound imaging system (Visualsonics, Toronto, ON, Canada) with an MS250 high-frequency echo probe located at the Dalton Cardiovascular Phenotyping Core laboratory. Speckle-tracking-based strain analysis of B-Mode ultrasound images was performed in the parasternal long view (PLAX). Images were acquired at the highest frame rates possible (200–330 frames per second). Quantitation of strain and strain rate were performed in the longitudinal and radial axes. PLAX views were used for the evaluation of longitudinal and radial strain, longitudinal and radial strain rate, and global longitudinal strain. Strain analyses were conducted offline utilizing the manufacturer-supplied speckle-tracking algorithm (VevoStrain^®^, VisualSonics). Briefly, at least three of the highest quality B-mode loops were chosen, i.e., those with little gel artifact or obstruction from ribs as well as those that displayed the endocardial and epicardial borders throughout the cine loop. Initially, the endocardial and epicardial borders were traced with the cine loop stopped at the end diastole. Cine loops were replayed to confirm good border tracking over all cardiac cycles and tracking adjustments were made as needed. The final tracked images were then evaluated for strain measurements. Strain measures were averaged over the cardiac cycles yielding curvilinear strain and strain rate data. Global strain values, peak strain, and strain-rate measurements of control ZDF rats were compared to those of ZDF rats treated with NP. The speckle-tracking algorithm also calculated ejection fraction (EF), fractional shortening (FS), cardiac output (CO), and stroke volume (SV) in the PLAX view.

### Appendix A.5. Evaluation of the Effect of NP-6A4 Treatment on Healthy Rats

Outbred Wistar rats are a widely used model for healthy pre-clinical controls for several pathologies. Although Zucker lean (ZL) rats are usually used as healthy controls for ZDF rats, it is established that ZL rats are not healthy rats since they suffer from hydronephrosis, a common pathology shared between both ZL and ZDF rats [68]. Therefore, we used female Wistar rats as healthy controls to evaluate the effects of the four-week treatment with NP-6A4 (10 mg/kg/day) delivered subcutaneously (using the same approach described for the ZDF-F rats in this study). After four weeks of NP-6A4 treatment, echocardiography analysis did not detect any changes in any of the cardiac parameters. Table S1 shows the effect of the four-week NP-6A4 treatment (10 mg/kg/day) in healthy female Wistar rats on the same cardiac parameters that were modified by NP-6A4 treatment in ZDF-F rats.

### Appendix A.6. Histopathology and NT-ProBNP

Tissues from ZDF-F rats from all treatment groups (randomly numbered for blind analysis) were fixed in 10% neutral buffered formalin (NBF), embedded into paraffin blocks, and sections were cut at 4 µm thickness by IDEXX Laboratories (Columbia, MO, USA) and used for histopathology as described previously [1,2,3]. The heart sections were stained with Trichrome or Picrosirius Red or monoclonal anti-NT-ProBNP antibody (LS-C664126) and horse radish peroxidase (HRP) secondary antibody by established procedures at the IDEXX Laboratories (Columbia, MO, USA). The stained sections were scanned using the Aperio CS Slide Scanner by WSI Analytics Lab, Department of Pathology and Anatomical Sciences, University of Missouri, Columbia, MO, USA. Scanned sections were visualized using Aperio ImageScope (Leica Biosystems, Wetzlar, Germany). To determine interstitial fibrosis, after scanning Picrosirius-stained slides, an Aperio ImageScope was used to quantify 20.8 ± 2.7 (mean ± SD) interstitial images (15×) of the most fibrotic regions per animal, avoiding perivascular staining. The built-in algorithm Positive Pixel Count (V9) was used with the following parameters to determine percent fibrosis (hue value: 0.0, hue width: 0.154, color saturation threshold: 0.04). Lastly, positivity values (positive/total pixels) were averaged over all regions from a single group to determine interstitial fibrosis. The statistical significance of the difference between groups was determined using a Kruskal-Wallis test followed by pairwise Tukey’s HSD tests. To detect NT-ProBNP staining, a similar procedure was used (with the following parameters: hue value = 0.1; hue width = 0.1; color saturation threshold = 0.05). To detect any vascular damage within the ZDF-F heart tissues, scanned images of two non-consecutive trichrome stained sections from each animal were visualized at 20× magnification. Images of the tissue where any such damage was observed were captured.

### Appendix A.7. Quantification of Microvascular Damage in ZDF-F Rat Heart

For each animal, whole heart tissue sections stained with Masson’s Trichrome (MST) were generated by IDEXX laboratories. These slides were scanned by an Aperio CS Slide Scanner, which enables visualization of the image at different magnifications. These images were visualized and scanned at 10× magnification for the presence of any microvascular damage characterized by the pink staining as shown in the Figure 1G. Only the pink-stained areas next to blood vessels within the reddish areas of an MST-stained heart section that were a minimum of 60 µm away from any of the cut edges of the section were selected. The pen tool of the Aperio Image Scope software V12.1.0.5029 was used to outline each area with microvascular damage and the area (µm^2^) was determined by the annotation tool. The total area of microvascular damage within each tissue section was calculated as the sum of all areas of microvascular damage within that tissue section. The total area of each whole heart section was calculated as follows. First, the periphery of the whole heart tissue section was outlined by the pen tool and the area was determined by the annotation tool. Next, areas corresponding to the white regions within the tissue section were subtracted using the negative- pen tool from the area calculated by the pen tool. The total area calculated this way was used as the denominator to calculate the percentage of microvascular damage in each heart tissue.

### Appendix A.8. Capillary Density and Cardiomyocyte Hypertrophy

To determine cardiomyocyte size and capillary density, heart sections were deparaffinized in xylene (Fisher Scientific, Waltham, MA, USA), rehydrated in an ethanol series and 1× PBS buffer, followed by a heat-mediated antigen retrieval step in sodium citrate buffer. To block non-specific binding sites, sections were incubated with blocking buffer (10% donkey serum, 1% BSA) for 2 h at room temperature, followed by incubation with wheat germ agglutinin (WGA) conjugated to Alexa Fluor 647 (Life Technologies, Carlsbad, CA; 1:200, 5.0 μg/mL) and *Griffonia simplicifolia* isolectin B4 (IB4) conjugated to Alexa Fluor 488 (Life Technologies; 1:200, 5 μg/mL) for 4 h at room temperature. Sections were thoroughly washed and slides were mounted using ProLong Gold Antifade Mountant with DAPI (Invitrogen, Waltham, MA). Imaging was performed using a Leica SP8 Falcon inverted confocal microscope at 63×. Confocal microscopy images were then visualized and analyzed in ImageJ 1.53n (National Institute of Health, Bethesda, Maryland). Cardiomyocyte size was quantified in the mid-left ventricular sections by manually tracing the outline of cells to measure area. A minimum of 200 cells per animal from 5 independent fields of view were analyzed for each group. The same fields of view were analyzed for capillary density by manually counting the number of IB4 puncta in the entire image.

### Appendix A.9. Quantification of LC3, RUBICON and AT2R by Immunohistochemistry and Immunoblotting

Five-micrometer heart sections from the saline-, NP-6A4-, and NP-6A4 plus PD123319-treated ZDF rats were deparaffinized in xylene (Fisher Scientific), rehydrated in an ethanol series and 1× PBS buffer, followed by a heat-mediated antigen retrieval step in sodium citrate buffer. Following rehydration, sections were incubated with wheat germ agglutinin (WGA) conjugated to Alexa Fluor 647 (Life Technologies; 1:200, 5.0 μg/mL). After washing, tissue sections were permeabilized with 0.2% Triton-X (Thermo-Fisher, Waltham, MA, USA) in PBS and subsequently blocked in blocking buffer (10% donkey serum, 1% BSA) for 2 h at room temperature before incubating with anti-LC3 antibody (MBL, Woburn, MA, USA; M152-3; 1:100) overnight at 4 °C followed by fluorescent anti-mouse secondary antibodies (Jackson Immuno, Grove, PA, USA; 715-585-151; 1:200). Sections were then thoroughly washed and slides were mounted using ProLong Gold Antifade Mountant with DAPI (Invitrogen). Imaging was performed using a Leica SP8 Falcon inverted confocal microscope at 63×. Confocal microscopy images were visualized in ImageJ software 1.53n (National Institute of Health).

For immunoblotting, the heart tissues from the saline-, NP-6A4-, and NP-6A4 + PD123319-treated rats were flash frozen in liquid nitrogen and stored at −80 °C in a freezer until ready for processing. The tissue was ground into powder and re-suspended in a RIPA Buffer 2× solution (Boston BioProducts, Ashland, MA, USA; #BP-115X) supplemented with protease inhibitors (Boston BioProducts) per the manufacturer’s instructions. Upon sonication and homogenization on ice, proteins were extracted by constant agitation for 30 min before centrifugation. Protein concentration was measured by s Bradford assay (BioRad, Hercules, CA, USA), and 40μg of total protein was loaded for electrophoresis and transferred to the polyvinylidene difluoride membrane. The membrane was processed for immunoblotting using antibodies against LC3 (CST, 3868, clone: D11; 1:1000), Rubicon (MBL International, PD027; 1:1000), p62 (Progen, Heidelberg, Germany; GP62-C; 1:1000), AT2R (Abcam, Cambridge, UK; ab92445; 1:1000), and Actin (Sigma, St. Louis, MO, USA; A5316, clone: AC-74; 1:5000) and imaged with a ChemiDoc XRS molecular imager (BioRad). Quantification was performed using ImageJ software 1.53n (NIH). Significance was determined using a ANOVA with Tukey post-test.

### Appendix A.10. Cardiac Proteome Analysis

Cardiac proteome analysis was performed at the Gehrke Proteomics Center at the University of Missouri-Columbia according to established procedures [69]. For DIA (data independent analysis), a spectral library (peptide MS/MS acquisitions matched to individual peptides) was used since the spectral library approach results in improved proteome coverage and quantitation of peptides/proteins [70]. To generate the spectral library, 6 µg of purified peptides from each sample were combined and fractionated by high pH reversed-phase peptide fractionation kit (Pierce/ThermoFisher). Eight fractions were collected and they were analyzed by the timsTOF DDA-PASEF acquisition mode using 90 min LC gradient. DDA-PASEF: Peptides were analyzed by mass spectrometry (MS). A 1 µL injection was made directly onto a C18 analytical column (20 cm long × 75 µm inner diameter pulled-needle analytical column packed with Waters BEH-C18, 1.7 µm reversed phase resin). Peptides were separated and eluted from the analytical column with a gradient of acetonitrile at 300 nL/min. The Bruker nanoElute system is attached to a Bruker timsTOF-PRO mass spectrometer via a Bruker CaptiveSpray source. Samples were separated using the following LC gradient conditions: Initial conditions were 3% B (A: 0.1% formic acid in water, B: 99.9% acetonitrile, and 0.1% formic acid), followed by 10 min ramp to 17% B, 17–25% B over 30 min, 25–37% B over 30 min, and 37–80% B over 5 min (see-saw column wash: 2 min to 40%, 2 min to 80%, 2 min to 40%, and 2 min to 80%), ramp back (2 min) and hold (5 min) at initial conditions. The total run time was 90 min.

For generation of the spectral library, MS data were collected in positive-ion data-dependent PASEF mode over an m/z range of 100 to 1700, and an ion-mobility range of 0.6 to 1.6 1/k0. PASEF and TIMS were set to “on”. One MS and ten PASEF frames were acquired per cycle of 1.16 s (~1 MS and 120 MS/MS). Target MS intensity for MS was set at 10,000 counts/sec with a minimum threshold of 2000 counts/s. An ion-mobility-based rolling collision energy was used: 20 to 59 eV (1/k0 0.6 to 1.6). An active exclusion/reconsider precursor method with release after 0.4 min was used. If the precursor (within mass width error of 0.015 m/z) was >4× signal intensity in subsequent scans, a second MSMS spectrum was collected. The isolation width was set to 2 m/z (<700 m/z) or 3 (800–1500 m/z). For protein identification and quantitation, the individual samples were lyophilized and re-suspended at 1 µg/uL in 5% acetonitrile and 0.1% formic acid. The same injection volume and LC gradient were used (as described above). PASEF and TIMS were set to “on”. MS data were collected in positive-ion data-independent PASEF mode over an m/z range of 400 to 1200 and an ion-mobility range of 0.57 to 1.47 1/k0. A total of 64 DIA-PASEF windows were used (25 m/z steps and 0.18 ion-mobility steps) with two collision energies based on ion mobility.

The DDA (Data Dependent Analysis) data were searched against UniProt-Rat (29,936 entries, reviewed and unreviewed) and the false discovery rate (FDR) was determined by simultaneously searching a reversed-sequence decoy database. The Spectronaut algorithm (V14, Biognosys Inc., Zurich, Switzerland) was used to generate a spectral library with the following parameters: trypsin as the enzyme, two missed cleavages allowed, specific; precursor and fragment mass error = dynamic (examines data for match tolerances); carbamidomethyl-Cys fixed modification; oxidized-Met and protein-N-terminus acetylation as variable modifications. The spectral library was generated using the following data filters: spectrum, peptide, and protein FDR 1%; min 3 and max 6 best fragments per peptide. A total of 60,758 precursors, 44,731 peptides and 5991 protein groups were included in the library.

For DIA-PASEF quantitative data analysis: precursor Q-value (precursor FDR), protein Q-value (protein FDR) was set at <1%. MS2 (MS/MS fragment intensity) area quantification was filtered based on Q-value. The final protein group (PG) quantity was cut off by 23 (1% quantile). A protein was identified in at least three samples per group. On average, >3700 proteins were identified in each sample. A total of 4520 proteins were identified in all samples. The global sum normalization method was applied (i.e., all samples were normalized to the sum of all peptide intensities). Then a one-way ANOVA was applied for the three groups (saline-, NP-6A4-, or NP-6A4 + PD123319-treated groups). For Ingenuity Pathway Analysis (IPA), differentially expressed proteins between saline- versus NP-6A4-treated groups (Table S2) and NP-6A4- versus NP-6A4 + PD123319 (NP + PD)-treated groups (Table S3) were identified via a pairwise comparison using a Student’s *t*-test. Differentially expressed proteins (*p* < 0.05) from each pairwise analysis were uploaded to IPA software Version 90348151 to identify IPA-predicted canonical pathways that had a Z score above 2. In the diseases and functions analysis, networks that had a Z score above 1.5 were selected.
