# Supplementary material for: Cardiovascular Protective Effects of NP-6A4, a Drug with the FDA Designation for Pediatric Cardiomyopathy, in Female Rats with Obesity and Pre-Diabetes
Source: Cells. 2023 May 12;12(10):1373. doi: 10.3390/cells12101373 (PMC10216951; doi:10.3390/cells12101373)
Supplement: Supplementary file 1 [file cells-12-01373-s001.zip › Table S1.pdf]

Table S1. Effects of 4-week NP-6A4 treatment on cardiac parameters of healthy female Wistar (Wistar-F) rats.

Cardiac Parameters	Wistar-F Saline (N=8	Wistar-F NP-6A4 (N=8)	Wistar vs. ZDF (P value)
Heart rate	407±11	428±13	0.28
E/A	0.96±0.08	1.06±0.8	0.08
Stroke Volume (SV) (µL)	202±12	196±10	0.73
Ejection Fraction (EF)	83±0.58	80±2.9	0.33
Cardiac Output (CO)	85.5±5	87.5±5	0.81
Radial Strain (Pk%)	43.28±2.4	47.80±3.4	033
Radial Strain Rate (Pk1/s)	7.±0.43	9.0±0.66	0.18
Isovolumic Relaxation time (IVRT (ms)	15±0.7	14±3	.40
Isovolumic Contraction time ((IVCT) (ms)	7.74±0.5	11.4±0.7	0.90
Systolic Time (Syst T) (ms)	86.3±2.5	82.5±2.2	0.3
Myocardial Performance Index (MPI)	0.44±0.06	0.4±0.01	0.76
