# Supplementary material for: Cardiovascular Protective Effects of NP-6A4, a Drug with the FDA Designation for Pediatric Cardiomyopathy, in Female Rats with Obesity and Pre-Diabetes
Source: Cells. 2023 May 12;12(10):1373. doi: 10.3390/cells12101373 (PMC10216951; doi:10.3390/cells12101373)
Supplement: Supplementary file 1 [file cells-12-01373-s001.zip › Table S2 revised.pdf]

Table S2: Differentially expressed proteins in ZDF-F rats treated with saline and NP-6A4

Accession	Genes	Saline	Saline	Saline	Saline	Saline	Saline	NP-6A4	NP-6A4	NP-6A4	NP-6A4	NP-6A4	T test NP-6A4 vs. Saline	NP-6A4/ Saline: Fold Change
A0A0G2JZJ5; A0A0G2K2U0; Q5PQL5	Ptdss1	243.1380646	187.6782376	224.508941	234.0242698	249.6552791	253.8852773	281.2819284	270.0130149	307.2857872	305.0527357	305.2303469	0.000862	1.265453
F1LWY1	Hfm1	640.5015016	616.3068788	645.5790409	588.4863495	643.5584876	661.3142187	505.9799701	382.0779641	512.3730973	489.8102338	397.2856754	0.001888	0.723186
P32089	Slc25a1	110.9069581	127.4280612	145.3234936	132.3268632	126.9845407	146.7939139	163.9367957	149.6371166	193.2498269	178.3010967	175.2318484	0.002333	1.307262
A0A0G2K3I5;D4A0T1	Dus4l	0.100186708	0.07815463	0.095494021	331.7499577	53.82028036	0.105369985	247.8866574	432.8654803	390.4766933	277.5227213	331.3598444	0.002722	5.223828
D3ZEH9;Q5XIQ4	Mgrn1	106.2893974	66.09897753	99.53628454	62.90444536	109.9178873	91.48601499	39.01250347	0.077969763	55.50051031	39.79042467	55.40986407	0.004149	0.424721
Q9QWJ9	Nrp1	355.1210636	332.2660078	355.8655742	400.0531432	317.1880621	410.817499	455.824561	415.6524084	409.932094	425.779901	451.4415813	0.004197	1.192992
D4AE06	Fkbp15	92.58781484	122.7923526	197.1656612	58.59811751	232.0112234	104.6840976	313.96823	202.0336276	327.2668968	421.7891578	271.7945521	0.005207	2.282908
Q5M8B4;Q9WTS8	Fcna;Fcn1	131.6344439	124.6004416	180.8490151	0.103804397	174.7493981	157.1469619	65.72937789	0.077969763	0.105148197	0.103324622	0.103353746	0.006466	0.103166
Q9JLZ1	Glrx3	994.972703	1096.68347	939.3735889	1069.779073	1081.250769	1234.027355	1264.475363	1186.221616	1342.926668	1204.296404	1227.287257	0.006925	1.1643
B2RYW9	Fahd2	2677.732417	2251.009224	2705.535858	2783.457379	2248.132783	2643.209158	3051.542773	2946.079873	2787.388347	2938.138055	3049.184059	0.007165	1.157927
A0A0G2JU46; Q6AY61	Prss23	122.096214	96.60163738	84.72028578	149.3577789	97.93552101	107.2465019	141.6920205	164.6122817	191.5562505	137.1982163	149.0960427	0.007431	1.430161
D4A7D7	H6pd	53.96117315	0.07815463	0.095494021	0.103804397	89.06032304	0.105369985	92.4625053	141.3584923	92.5880368	53.76633483	97.90777218	0.007671	4.000575
A0A0G2K4M8	Acot3	114.3555541	129.4625191	104.1411215	146.985908	131.8180109	108.8359027	151.6802194	132.2913665	152.7564598	161.5449633	157.4233873	0.007869	1.232785
A0A0G2K059; M0RDI5	Mcu	110.717006	157.2345431	117.6581241	152.4481202	137.0330521	160.3113896	160.695343	188.3307535	184.6986421	165.0691724	171.4437513	0.008814	1.250039
A0A0G2JZG3; O70215	Klrk1	0.100186708	717.0168755	891.6498342	1062.652025	1113.995489	1195.839611	0.108550851	548.4715008	0.105148197	0.103324622	0.103353746	0.009122	0.13223
A0A0G2JW28; D3ZCH7; G3V9D7	Add3	149.1516471	223.4848666	211.5919378	209.5073394	200.2721717	184.2025577	130.4862438	136.9595586	169.1067189	158.0262251	165.0319742	0.00926	0.773659
A0A0G2K9N0; B1WBX6	Smap2	68.18428889	31.48111022	65.58955601	0.103804397	53.45970026	0.105369985	108.924657	70.96812904	86.03675508	80.79790447	84.09463294	0.009331	2.36149
Q8R560	Ankrd1	475.0378392	401.9193419	493.5162946	566.4037761	380.6740788	458.5912815	621.610047	543.4952654	518.9233602	580.3977505	672.2411779	0.010613	1.269388

Q66HF2	Tm9sf1	0.100186708	26.43679688	25.97018339	57.72845711	35.30533091	33.91250707	0.108550851	0.077969763	0.105148197	0.103324622	0.103353746	0.011187	0.003332
Q9Z2S9	Flot2	356.5366045	373.1337859	373.7817481	344.3916783	377.906833	342.0759962	343.0105517	292.6794492	324.8110857	275.0657974	324.7787391	0.011719	0.863729
Q32KK0	Arse	109.1788153	113.4028137	153.9687945	0.103804397	0.097116934	0.105369985	201.9948704	172.2837659	164.1079919	145.624338	166.4878464	0.012049	2.708187
D3ZTB5	S100a13	194.5437657	159.6593449	150.8747976	243.500967	127.5982641	140.1210335	281.5549287	186.4992564	257.0551573	253.374753	232.1654651	0.013296	1.429482
F1M4J0	Rictor	37.55031028	12.4761942	32.6925936	0.103804397	39.58331585	21.20964861	0.108550851	0.077969763	0.105148197	0.103324622	0.103353746	0.013326	0.004164
D3ZWQ8; Q5XXR3	Arhgef6	123.65158	121.3013493	112.7800475	209.6905367	175.3003043	132.5041146	70.53067349	131.5463374	51.71605524	95.28458853	44.28072712	0.014817	0.539322
A0A0G2KAW7; Q5XI72	Eif4h	291.7341062	268.6737599	272.9512469	343.2550506	421.0371783	343.9085233	389.8689539	379.5277666	383.4312056	458.2027626	469.6580575	0.014977	1.28599
A0A0G2JWM2; Q5RJQ4	Sirt2	285.8354792	236.6985992	269.2468902	303.7742798	237.3166302	265.1723231	309.3706205	290.9744794	301.3736077	299.9140637	345.8148285	0.015171	1.162006
Q9Z1P2	Actn1	31232.18877	31751.41125	26433.17026	26872.34302	33796.01023	27559.32609	27098.592	25156.04245	20737.44247	26504.60612	23053.97361	0.015463	0.827838
D4A6D9	Hs1bp3	101.9407018	85.97270434	106.6184785	111.1500914	107.8874579	109.4813935	157.1900993	148.6409486	110.1434505	159.770112	125.0232432	0.01626	1.349684
D3ZQ74;Q63321	Plod1	76.96182378	60.73883952	67.7089343	116.7703877	87.54985063	97.06598885	120.4924879	149.728059	145.2899527	90.96541404	119.0399435	0.016659	1.481107
P04550	Ptms	27.49505127	0.07815463	76.26850538	102.2694111	90.33801327	76.01160726	118.7304745	103.4929391	178.6712161	104.2856599	206.0240608	0.016878	2.29137
Q4KM64	Jagn1	240.3697524	207.9474362	302.3960333	300.2700895	66.15943689	335.1826036	171.608533	0.077969763	0.105148197	178.8360715	0.103353746	0.01727	0.289795
D4A4T0	Stub1	337.9027573	398.6396523	440.8869017	531.8817814	391.1463655	495.8659137	559.7243351	460.1399543	542.6135366	533.6026824	580.4551155	0.019459	1.237073
A0A0G2K6B7; F1LM79; Q8K4T4	Filip1	124.7438713	0.07815463	107.4189418	163.5787564	0.097116934	191.2971367	257.5770436	175.4327991	235.0628413	147.0966224	245.1604134	0.019546	2.166835
Q5M936	Tor3a	0.100186708	161.2707832	440.2157799	0.103804397	406.6577489	0.105369985	463.6390097	305.1771447	529.1426432	411.4094288	537.4355064	0.02012	2.673563
D3ZY71	NEWGENE_1586233	233.195769	143.5615733	189.9422939	161.2166425	218.0011098	282.8393369	294.0220774	349.1883013	255.4607347	312.3272394	229.9219337	0.020179	1.407198
P97521	Slc25a20	4993.921431	4278.72136	5281.440446	6549.17755	5492.338668	5867.052722	6663.577899	5882.804137	6128.903585	7231.300334	6665.807435	0.021093	1.204057
Q496Z0	Elp2	100.6560582	100.6402447	107.6523656	70.89153211	82.96121776	103.9515916	76.31525153	63.52347767	80.93306605	69.81804731	83.7771564	0.021739	0.792656
A0A0G2JXH2; A0A0H2UHS3; Q5PPN7	Ccdc51	1466.437813	960.6335472	1114.351111	1683.035354	1276.761906	1206.968673	1709.010841	1471.704876	1646.526941	1614.370287	1678.96016	0.021798	1.264199
J7PDL5; M0RAS6; Q5FVQ6	LOC103694226; LOC100910979;	68.28923605	37.77987059	65.99263997	0.103804397	48.85527788	62.23273243	85.79156225	112.7657087	112.4568842	104.3816833	47.23815886	0.02239	1.959943

	MGC108823													
Q9ET09	Mrs2	110.3674853	134.7153818	72.00461341	132.7830665	106.155599	151.7482776	88.1216804	61.70913047	107.9548019	69.28604438	65.53824655	0.023225	0.665653
P35859	Igfals	138.8720072	112.7619281	142.0136385	195.8997386	101.935467	139.9645285	215.9100208	178.3889662	248.0874934	163.0209528	173.914331	0.023253	1.413422
P23965; Q68G41	Eci1	38510.21696	41828.78216	36877.41102	46202.67619	38850.71523	43036.5922	43752.66706	50690.94484	47265.01416	42859.2794	46796.2993	0.023846	1.131797
A0A0H2UHE5; P20650	Ppm1a	133.1868823	128.9149025	146.7565888	175.4208461	176.8010852	181.4765659	211.6142488	180.8944064	173.5616068	181.7084789	200.5573958	0.024227	1.207358
D3ZT51	Exosc3	57.30753165	59.53619366	0.095494021	0.103804397	56.88601521	66.04921895	0.108550851	0.077969763	0.105148197	0.103324622	0.103353746	0.025533	0.002492
D3ZTN2	Cmc1	931.3974369	1312.113673	885.074362	1056.999424	1035.403444	971.4892011	1208.328655	1402.534599	1090.013164	1452.186954	1225.351519	0.025917	1.236032
Q4QR73	Dnaja4	436.7120205	444.987294	470.1764752	453.7657731	327.5693608	366.6156457	430.885692	582.73332	544.0355786	468.2163401	578.9357195	0.026374	1.250394
G3V7K2; O70535	Lifr	201.724552	239.8433164	131.2017994	215.5948711	37.54511392	131.8324806	34.21320171	48.55604575	120.5433126	36.42278077	82.56198974	0.026551	0.403821
Q68FW7	Tars2	105.3705097	83.26119684	102.2516335	51.84499492	162.7530798	196.9202242	168.5969161	189.2430696	189.335874	204.8508705	167.0359828	0.026626	1.570149
D4A9M6; O88884	Akap1	214.5661564	161.4956853	196.4201533	267.7579428	186.2158084	139.3865982	274.552458	248.0829245	220.8090482	259.1840986	250.3219503	0.0268	1.28966
P31399	Atp5pd	32131.91824	28165.02352	30875.52601	35457.11668	25753.7038	33384.77774	35090.94944	37603.81457	36486.6257	36181.1225	32283.77199	0.027385	1.147536
A0A0G2K466; D4A0Y4	Oxnad1	336.0147478	174.8709601	191.9091478	244.7832053	243.7559915	229.9478041	298.6100801	294.5269244	356.2198739	266.7576061	340.2939128	0.027906	1.314088
B5DFB2	Rbbp4	308.9446466	284.8624392	358.0870167	394.3770689	313.994922	395.4196153	404.4724356	362.0454362	453.1264459	385.263519	453.4978375	0.028387	1.201588
Q91XQ2	Epm2a	129.24226	130.0152399	122.9839893	116.1278826	114.8051871	116.4899381	136.1696021	151.2480388	127.6503061	129.3596099	156.0759059	0.028736	1.152042
D3ZYK9	Npm3	160.8774637	151.5616065	197.3850456	202.7775719	184.4793483	182.139984	134.1549614	118.5107559	163.1265916	140.9206522	173.0915825	0.028793	0.811479
A0A0G2JZV3; G3V8G1	Agtpbp1	96.81937174	68.70933334	89.24739975	0.103804397	66.98285471	108.6410592	61.30582223	0.077969763	0.105148197	36.32326129	0.103353746	0.029659	0.272933
A0A0G2JX77; A0A0G2K7S6; F1LSM3	Wdr44	0.100186708	0.07815463	0.095494021	0.103804397	47.91607283	0.105369985	50.11170208	0.077969763	74.65570135	61.25504446	73.77808832	0.029803	6.443391
D3ZCT5	Pald1	163.430271	165.4890296	192.6359754	195.7761921	189.9933009	209.2600051	220.5054274	212.5887176	259.2421614	209.0782312	199.020605	0.029943	1.182644
G3V6Z7; Q3ZU82	Golga5	42.23143164	77.17469756	81.88589716	1.038043971	51.89564282	135.5620645	46.94393645	0.077969763	0.105148197	0.103324622	0.103353746	0.030133	0.145722
D4A777	Fam114a1	37.19122357	34.35201028	60.47316687	14.58845999	40.268996	37.30305435	48.40687847	62.74508278	56.66430267	55.32778436	52.16676099	0.030544	1.473715
Q8VIE2; Q8VIE4	Pde7b	0.100186708	0.07815463	0.095494021	0.103804397	38.59553826	0.105369985	47.13206107	0.077969763	64.20767824	54.12925831	41.24298	0.030978	6.349978
D3ZSY8	Tbc1d10b	28.27458017	0.07815463	48.79193171	44.30842521	34.40175043	0.105369985	0.108550851	0.077969763	0.105148197	0.103324622	0.103353746	0.030984	0.003834

P04166	Cyb5b	2205.097463	2026.560805	2204.004803	2619.588099	1959.099771	2344.74919	1605.969025	1033.997075	2134.867797	1779.37991	1753.611542	0.03101	0.746262
A0JPJ0	Nmnat1	68.34381163	98.16935704	130.1002144	0.103804397	104.7368554	0.105369985	0.108550851	0.077969763	0.105148197	0.103324622	0.103353746	0.031652	0.001489
Q9JJ54	Hnrnpd	886.4719779	769.7382409	965.10073	493.6450605	817.3312925	644.7763918	564.5439955	627.8243894	535.9958874	540.2951338	536.1526053	0.032975	0.735357
D4A8L4	Dcaf6	120.163046	111.8859918	196.266456	0.103804397	124.785465	0.105369985	0.108550851	0.077969763	0.105148197	0.103324622	0.103353746	0.033327	0.001081
Q5PQJ6	Pycr3	261.6004343	220.3674346	234.7014006	395.6893861	482.2998907	281.5751093	524.6590114	351.3727301	517.0303846	372.2518942	519.510785	0.033598	1.461326
P60841	Ensa	96.77420548	246.1528575	414.9921335	0.103804397	102.5990337	205.6730426	29.47819135	0.077969763	0.105148197	0.103324622	0.103353746	0.033739	0.033613
D3ZRL3	Dusp23	279.8688544	334.0243439	282.968269	320.0405378	280.0445395	219.8037886	324.9824245	320.4977938	315.9790287	342.8097688	368.3468472	0.033833	1.16915
D3ZBS9; O54772; Q5U3Y2	Smarcd1 Smarcd2 Smarcd3	212.3172753	207.6450306	165.6382525	189.6102084	217.0650761	263.871373	256.3072988	0.077969763	0.105148197	0.103324622	0.103353746	0.034539	0.245223
B2GUZ6	Rtn4ip1	1656.434714	1559.836755	1700.304266	1924.213567	1817.577802	1866.292155	2351.370704	2272.059637	1821.955644	1920.130447	1978.664576	0.03487	1.179422
Q5M9I5	Uqcrh	4368.856657	2256.500656	2533.243054	2419.756777	1746.812134	5012.983719	4706.192455	4641.307746	4735.561103	4900.224201	3919.877803	0.034936	1.498722
A0A0G2JXC3; P05765	Rps21	598.4436915	732.9622994	552.6719963	169.9712579	513.1985052	390.3926426	433.7480052	233.1414405	170.1896798	271.6627508	200.4250968	0.03611	0.531167
D3ZSW0; M0R7C8	Olr390;	26064.26875	19345.19265	28297.1147	34439.87009	19069.57959	32860.52707	32473.5743	37969.20037	40613.91423	35040.28951	28853.05339	0.036142	1.311498
Q9QZP1	Gemin2	62.28990413	27.05154489	60.83344057	50.18677527	0.097116934	0.105369985	0.108550851	0.077969763	0.105148197	0.103324622	0.103353746	0.036181	0.002982
B2GV53	Slc25a32	200.6397667	227.0286094	93.67566978	76.4911898	127.1608852	55.4321511	84.74545842	0.077969763	32.65141444	66.68143176	59.15471445	0.036668	0.374119
A0A0G2K3Z9; Q63716	;Prdx1	1078.695685	875.1906552	946.7367818	1186.946766	994.4378691	949.1496582	1232.211962	991.0563139	1145.075291	1364.750384	1299.339151	0.036896	1.200254
G3V8R0	RGD1311703	123.4223396	119.9059524	119.0100721	124.1920948	134.0667741	135.6523596	75.91621087	78.24357188	117.7026458	102.7838772	117.9475031	0.037158	0.781637
A0A0G2JT25; Q920P0	Dcxr	169.3253361	221.4410352	189.3918219	195.0524948	325.946428	331.1900369	120.0255864	164.1532147	157.8403661	194.2782592	146.8136212	0.03741	0.656079
B0K013	Coa4	210.5563338	170.2245781	246.1688867	348.5814567	275.4225346	0.105369985	293.671129	290.3426406	392.0610399	368.7754092	374.1823534	0.038747	1.648874
D4A7I6	Washc4	65.21387131	86.83336407	64.45171042	92.69573789	75.62938271	80.0694675	75.28016245	116.623663	97.97888745	127.4465845	144.4228877	0.040066	1.450015
Q920F5	Mlycd	2813.051003	2352.869359	2257.502904	2444.97116	2321.666615	2684.175855	3361.132396	2913.591458	2562.699279	2918.254119	2683.981225	0.040356	1.16494
Q4QR80	Mrps25	319.2274816	261.8058694	353.8373754	344.8546292	321.5425272	332.8074057	376.0088774	319.455762	375.0612257	410.7583525	368.2911457	0.040497	1.147572
P61943; Q569D2	St3gal6	82.73905449	86.74344021	101.842262	121.171185	119.8645636	0.105369985	161.9631002	98.72154438	149.133872	145.465337	130.4586589	0.041223	1.605748

A0A0G2K151; P02650	Apoe	1227.5758	834.0984163	714.0508302	543.7928671	1102.781992	957.3950653	508.302941	759.7276797	675.5747284	533.5439065	592.6621367	0.041263	0.684755
P07896	Ehhadh	293.2149236	328.9452708	298.9678306	247.5865385	207.7110064	308.9005292	493.3802136	399.4243804	303.3725723	327.5062596	364.8914295	0.041302	1.344719
A0A0G2K0W9	Psma7	748.1773865	729.6706843	752.4696882	533.9937928	706.1484667	650.0317713	555.9147859	606.7839748	596.2151148	512.2606045	658.4297262	0.041355	0.853181
Q63041	A1m	5106.989079	6930.917418	4910.135632	4631.192627	5421.63204	4499.868422	3190.915864	5264.370669	4594.425834	3001.587092	3011.911609	0.04178	0.726201
D3ZXI2	NA	757.1474112	1292.174416	1046.757152	834.3445677	1531.247395	1138.754727	568.1551796	819.8274051	734.7398425	973.2947356	762.6439757	0.041985	0.70153
Q03336	Rgn	105.637395	54.15703455	0.095494021	133.5665096	65.74119657	0.105369985	0.108550851	0.077969763	0.105148197	0.103324622	0.103353746	0.042922	0.001664
Q99M64	Pi4k2a	317.5853833	330.5479321	382.0436071	258.9320347	388.979758	325.2214614	444.3748445	401.3970382	453.5105153	377.1134615	336.972166	0.04313	1.206025
Q6IRG9	Ap3m1	170.0706367	133.7198333	62.32795347	46.74322168	45.40021802	0.105369985	168.4593061	194.2068725	78.97468569	149.0213778	175.0678034	0.043322	2.004672
Q6AYS7	Acy1a	477.6487841	389.3333172	483.5875742	561.1183303	472.1359254	446.9896179	437.5885613	382.7849696	328.8256688	432.04465	419.8376911	0.043372	0.848271
P02767	Ttr	2556.116124	2917.815901	2467.096193	3112.350562	1514.776875	2026.95064	2710.469427	3642.707344	2875.032809	3139.802189	3192.853084	0.044414	1.279404
D3ZKQ4	Rabl6	112.4842537	144.9738805	144.6878965	128.0770539	70.16516218	129.4457707	174.3299567	211.2445435	151.5187511	121.7927793	170.2269446	0.044789	1.363235
A0A0G2JW03 Q5XIM8	Lpin1	124.444814	77.38881305	151.9662265	236.4908755	192.1122064	111.2782672	219.8388772	174.9953375	233.2323263	219.4527258	211.8502939	0.045082	1.42248
F1LZB0	R3hcc1	133.6246183	160.5368434	185.4922107	0.103804397	0.097116934	0.105369985	182.2904462	142.0769787	184.3695567	171.3016166	200.031007	0.045371	2.200358
D4A4W6	Slirp	770.8015802	773.915583	686.7011537	772.7512555	607.7111121	762.8711658	752.9414706	906.8328849	823.7554542	773.9145393	835.6270242	0.046224	1.122735
D3ZZE3	Armh3	54.70985997	40.62485366	43.12743478	29.55389122	55.30816141	54.05042817	61.11443875	61.89880819	73.8832385	79.0817581	128.6117642	0.046336	1.750369
A0A0G2K602	Noa1	46.11770242	63.57184944	54.67896413	114.9806425	78.79408536	75.7362764	86.01941866	0.077969763	0.105148197	0.103324622	36.28504074	0.04683	0.339055
P29419	Atp5me	39568.50169	40389.05177	32711.35007	18917.10652	30660.60707	30370.78251	27918.42866	17143.09297	24585.92604	22391.76953	25305.89226	0.047074	0.731056
Q9Z0G8	Wipf3	328.1866828	259.1826758	318.0447196	235.5354968	215.6101745	413.1109233	411.5827681	319.2426824	375.0342711	367.3808358	402.1699347	0.047685	1.271701
F1M1H0	Dera	235.5710451	219.1753667	245.5414786	274.1947785	225.7209844	243.3615594	289.0511918	247.4632686	246.4550457	274.105422	285.4161583	0.047902	1.11598
D3ZN76	Sec16a	47.32034117	52.50415524	55.72107309	93.2550392	156.3140148	273.94868	239.7463042	71.78344105	388.4197445	410.2435898	315.0933311	0.048066	2.518681
D3Z831	Ndrg4	245.0011557	213.5570009	0.095494021	0.103804397	245.2444134	268.6164148	329.7013124	257.3010263	238.3856083	318.1091678	340.9365619	0.048631	1.831469
G3V9Y9	Ap3s1	50.1386656	79.81566003	87.84126865	0.103804397	105.7105884	66.62184747	59.70580446	41.1217658	0.105148197	0.103324622	0.103353746	0.049144	0.311013
P20595	Gucy1b1	59.44876343	46.32584788	74.36168493	111.2216216	86.51264842	109.0965381	103.1179831	109.7738618	151.5845008	92.93875256	114.5535036	0.049438	1.409463

Q5I0M1	Apoh	514.707379	281.2082189	528.3776156	513.8922716	320.029254	501.0373008	853.8620923	725.4559144	566.6447515	521.0886983	504.9553742	0.04953	1.431383
Q6AXT5	Rab21	244.1731662	135.9371084	232.5721794	222.6571055	210.6605836	233.4457094	343.968289	292.7055042	262.2356666	260.7399275	215.4012969	0.049564	1.289668
B5DEI0	Pcyox1l	140.1618255	161.1970362	143.6604352	199.0571118	175.4304732	190.2356228	204.0022562	248.8493448	159.5420451	224.8104518	212.305526	0.049821	1.24726
A0A0G2K5T6; Q5RJK9	Polr1c	40.27740024	94.12281926	45.49385009	0.103804397	77.83481557	65.84328153	62.18476677	0.077969763	0.105148197	0.103324622	0.103353746	0.050	0.23199
F1LZ43	Dip2a	130.9828543	80.14939968	217.450622	161.9401815	0.097116934	216.0334545	0.108550851	75.4744413	0.105148197	0.103324622	117.6331333	0.050	0.287744
O35568; Q6AXN2	Efemp1	180.7227512	186.494577	207.6997871	240.3906681	243.048761	224.4753692	166.5909879	162.2065999	206.8547869	161.3531215	203.3042279	0.050	0.842177
