# Supplementary material for: Cardiovascular Protective Effects of NP-6A4, a Drug with the FDA Designation for Pediatric Cardiomyopathy, in Female Rats with Obesity and Pre-Diabetes
Source: Cells. 2023 May 12;12(10):1373. doi: 10.3390/cells12101373 (PMC10216951; doi:10.3390/cells12101373)
Supplement: Supplementary file 1 [file cells-12-01373-s001.zip › Table S3 Revised.pdf]

Table S3: Differentially expressed proteins in ZDF-F rats treated with NP-6A4 and NP-6A4+PD123319 (NP+PD)

Accession	.Genes	NP-6A4	NP-6A4	NP-6A4	NP-6A4	NP-6A4	NP+PD	NP+PD	NP+PD	NP+PD	NP+PD	NP+PD	NP-6A4 vs NP-PD T test	NP+PD/NP-6A4: Fold Change
P97612	Faah	32.7787904	24.07917089	31.45792401	33.29173854	32.5660915	0.122760455	0.10093384	0.104457878	0.097552021	22.18941499	0.100763788	0.000358683	-8.14445
Q91ZW1	Tfam	1032.031247	1149.392134	1363.368186	1073.67665	845.5652969	552.4325388	807.6105637	339.056125	364.5785583	291.973586	722.662649	0.000398368	-2.13001
A0A0G2K0W9	Psma7	555.9147859	606.7839748	596.2151148	512.2606045	658.4297262	910.0002378	759.0537107	742.3716966	772.7784221	716.0146785	769.1016924	0.000406316	1.3282
M0RCH6	Chmp4b	434.0543313	291.5049298	392.1614454	388.4858233	448.2432231	233.7201836	265.051808	159.279488	128.843198	203.5734995	296.3065159	0.000415782	-1.82265
P23965; Q68G41	Eci1	43752.66706	50690.94484	47265.01416	42859.2794	46796.2993	40122.3319	40966.09256	37227.12706	40942.06108	35815.95446	35671.04623	0.000521141	-1.20322
G3V9U2	Acaa2	50877.44455	62012.00212	73178.47931	76043.09587	64520.50995	46300.90398	39628.82169	23959.27297	24783.70821	22134.70606	44540.10561	0.000527515	-1.94667
G3V8M4; P10817	Cox6a2	1864.186747	2045.534253	2506.043756	1377.044022	2273.484166	1095.072264	1461.703862	692.9491067	1125.975147	533.4297256	988.3125634	0.000610572	-2.04827
D3ZV63; D3ZV64	Odr4	50.91810656	35.31298813	42.4173565	50.18586848	68.21489189	0.122760455	26.08907436	0.104457878	0.097552021	0.10510375	27.29969716	0.000650967	-5.50848
O35115	Fhl2	4006.818882	2010.27654	4007.935579	3332.507908	3520.422891	1322.525418	1956.318243	797.5351982	599.1624399	616.550889	2625.986241	0.000888874	-2.55789
A0A0G2JZH8	Pdhx	3209.539649	3506.125868	3834.823551	2131.246917	3123.677482	1319.810513	1828.553891	1226.730649	1907.854475	1263.528236	2555.663645	0.000905806	-1.87747
Q03344	Atp5if1	1413.793472	1252.625451	831.8794103	993.7178713	1060.219685	350.9525409	825.9613141	247.7432258	160.3466835	316.0550401	749.120708	0.001003487	-2.51405
F1LXT8	Dnah6	4053.512179	3611.794694	5566.927031	3758.229821	4360.680269	1814.960583	2805.270528	597.8838306	444.2128082	667.4471105	3223.374543	0.001116222	-2.68198
D3ZYX8	Cox7a2l	2055.065441	1528.484033	2446.384477	2062.033421	2907.761514	1294.354652	1291.50898	1117.841472	471.5255878	788.2865291	1631.674714	0.001235817	-2.00141
F1LV13; F1M3D3	Hnrnpm	598.7916431	529.4035061	468.197634	514.421788	479.2933783	657.362008	694.7454124	620.4778834	638.8579672	716.1298281	582.5670961	0.001457244	1.258036
P20070	Cyb5r3	215.3123418	264.8213203	246.1438016	214.0118179	209.9833628	344.9812682	263.0272787	296.7942934	284.8010825	328.44367	298.4592779	0.001460522	1.315997
D4A4L6	NA	154.669873	116.4698197	155.3257525	127.6065314	29.61024863	0.122760455	0.10093384	0.104457878	0.097552021	0.10510375	15.99460622	0.001605581	-42.3843
Q68FU7	Coq6	1592.524726	1393.714079	1579.331185	1488.197326	1748.750686	995.9838816	1168.427233	787.7793144	1057.617641	1043.656246	1204.996898	0.001809393	-1.49606
P07895	Sod2	21982.27662	20065.51084	22050.18074	21450.95444	20223.67365	13546.29735	17281.09171	9064.350737	8931.503875	11988.35954	18709.94615	0.001871195	-1.59613
Q5XIJ3	Idh3g	2383.419176	1578.942139	2402.299504	1997.367746	2056.18998	982.1477803	1497.468225	808.6094292	891.3571189	838.9312816	1934.783063	0.001977404	-1.79798
Q9WUL0	Top1	73.43619264	60.06823911	113.6220002	87.62657478	145.6428487	0.122760455	64.1853653	0.104457878	0.097552021	0.10510375	53.0425572	0.002023687	-4.89959
F1M8K0	Dag1	586.5416426	544.1899215	465.3587802	497.9352864	550.6144338	613.1508611	654.6635151	648.008757	659.8657091	672.7216073	559.3233429	0.002027093	1.199827
B2RYS2	Uqcrb	9358.632098	5768.364475	8932.006658	8608.839032	9524.770388	4106.271595	6350.792698	4152.386313	3971.285132	3794.351097	7671.297751	0.002199556	-1.6851
A0A0G2K3I5; D4A0T1	Dus4l	247.8866574	432.8654803	390.4766933	277.5227213	331.3598444	0.122760455	318.9551852	158.4440928	0.097552021	0.10510375	0.100763788	0.00223003	-4.21939
Q925F0	Smpx	2604.320953	1786.747927	2930.712065	2238.53072	2850.495818	2345.717181	1393.736527	917.4598428	813.1281861	878.2694265	1544.24011	0.002574023	-1.88697
D4A732	Mvb12b	100.3295599	82.0146108	99.26882042	95.40670475	124.4251931	208.5910607	212.0842897	254.4440641	218.7445837	210.1798835	142.0638416	0.002591754	2.070862

P25113	Pgam1	15779.38948	20670.55316	15243.63091	15204.25956	16300.40077	6423.5703	14565.41944	4059.165563	6886.475802	5623.008032	12657.96479	0.00297971	-1.98818
P46413	Gss	371.7120949	383.6935857	356.0023126	273.3924797	401.6124149	521.5965046	478.7972029	458.7495263	451.2294802	492.6607417	369.0580755	0.003256064	1.293137
I6L9G6	Tardbp	829.7850578	799.3522522	723.2590893	655.6534421	741.1672892	918.1914862	825.0969942	1014.529864	929.6627204	1058.575731	821.6254321	0.003312884	1.237521
B5DFN4	Pfdn5	342.2335888	180.1790252	167.6671243	267.4735125	247.0891808	418.5153355	335.2475851	375.4550476	406.8328911	375.2898624	282.0890369	0.004118178	1.517345
Q7TQ84	Fyttd1	90.90491918	59.83446426	136.8277336	62.83550789	95.06120726	0.122760455	41.21551252	56.23293326	0.097552021	0.10510375	69.15257067	0.004321474	-3.20235
P16086	Sptan1	3097.314872	2541.024291	2741.876069	2832.654866	2995.916254	4116.573463	3266.950439	3946.975099	3873.173426	4501.588995	3197.283463	0.004337845	1.343215
P36201	Crip2	6889.85925	5776.06475	8112.627009	6496.871641	6990.734355	4364.831009	5366.311227	5259.40673	4194.662588	5006.494479	6164.716262	0.004608395	-1.35455
P43278	H1-0	977.7972303	760.0885945	1594.499377	866.901276	987.2230745	285.3983716	773.9111424	378.3277987	429.5264777	379.6413014	725.4420788	0.004657403	-2.09398
Q8R4A1	Ero1a	289.9133906	295.0953031	231.4880497	227.4241535	301.2020174	289.9317382	389.4822747	351.05807	379.1698018	378.3131027	302.0198362	0.004741582	1.294786
A0A1B0GWW9	Picalm	0.108550851	0.077969763	0.105148197	0.103324622	0.103353746	295.62919	155.9323254	224.2545803	236.2070444	146.9484371	162.0569879	0.004859165	2041.797
A0A0G2K904	Sltm	245.112661	166.3774992	289.4017052	249.366541	264.5654199	160.6320821	204.6244941	163.190777	144.0580891	172.8132518	170.6125924	0.005014627	-1.43493
Q9JKW1	Timm22	187.1028849	200.9334182	184.7236713	116.8154732	206.88305	285.351692	224.5127698	226.8011076	286.9783491	309.6013016	209.5011169	0.005020295	1.434112
P39069	Ak1	582.0822061	1018.151092	638.5164066	700.4197934	709.8793775	1236.381356	995.8511281	977.9158598	1199.866397	908.9574891	895.738539	0.005026217	1.419254
F1M7W7	Nae1	122.8853157	140.5766294	142.0293253	152.230533	108.4552131	187.2196784	164.634835	189.4666927	161.3881752	161.3928576	161.8606449	0.005073537	1.283396
B1A2U8;D2XRB8;Q6IMY1	Mtus1	126.702865	84.04828365	120.7136232	125.399705	139.4057774	237.4054885	195.1064403	232.6841314	216.7029813	257.0045856	117.4111466	0.005183037	1.755796
A0A0G2JW60;G3V7L1	Utrn	149.1519432	194.5110017	170.236786	115.3804913	149.1126334	264.3246744	219.8490153	209.5907414	190.7343404	216.4121593	176.0129479	0.00522869	1.367052
D3ZQM0	Sf3a1	100.2591152	89.77285561	60.88950382	74.47083219	64.79265531	92.95972541	113.3985671	98.25754055	108.7310275	134.4914312	113.6573818	0.005483683	1.412782
F1M944	Casq2	12704.10889	12645.24273	10105.00976	11356.61408	11002.76126	14628.11068	13429.98766	14485.19233	15689.28008	12277.20805	12609.99571	0.005580388	1.198097
P10760	Ahcy	910.7311039	682.9288911	822.9330854	800.1942085	885.8431047	474.5014535	788.5559748	486.056138	406.8244957	622.3698929	690.3056278	0.005636884	-1.41934
M0RDJ3;Q63184	Eif2ak2	128.4173907	122.9952902	103.7066292	121.2599092	144.3164997	188.5487904	154.8292855	155.4339634	157.481346	163.6261679	125.8248983	0.005836707	1.269737
A0A0U1RRU5;D4A6C6	Tab1	104.4004984	98.63459344	168.3561164	101.2244536	73.68261353	0.122760455	99.06081314	0.104457878	0.097552021	0.10510375	0.100763788	0.005847526	-6.58247
B0BNJ9	Tmem14c	447.8101239	360.5146419	502.740941	547.5554164	515.6048346	829.942948	714.3517984	692.4432664	639.2013402	552.3504213	538.3098741	0.005980017	1.392243
A0A1W2Q627;P63074	Eif4e	157.3180526	230.4674164	187.1533943	160.2488333	199.752468	271.3095039	292.7067191	271.806648	302.2579464	338.9067983	187.1285147	0.00608266	1.483264
A0A0G2K777	Rhot1	310.0594321	228.9546822	204.0232173	256.2877108	303.8081605	303.572486	397.0609682	308.2727377	465.9776143	418.9988196	376.7579812	0.006342021	1.452039
P38718	Mpc2	12197.92271	8410.725476	9888.805964	12062.33336	8510.568352	5621.925345	8458.645137	6465.327539	7953.071396	5969.979221	9041.960798	0.006478154	-1.40848
P62912	Rpl32	845.3814908	1489.064951	747.2091198	972.1962808	1235.457023	346.8844524	686.1827926	533.9379998	419.8266785	525.345288	625.0886276	0.006615259	-2.02315
A0A0G2K5P5	Myom1	12178.21607	12639.68891	8781.312041	10920.55088	11846.34887	15350.64108	12882.20966	15173.46467	14864.9474	13551.96668	11821.91563	0.00688087	1.236635
G3V6K6	Egfr	214.2330943	141.9538897	235.5497068	185.9409164	230.39557	265.1018552	212.7490244	278.4087123	335.0352528	278.8515906	255.6586381	0.0069929	1.343987
D3ZCZ9	LOC100912599	958.6620952	476.2553868	1048.900052	764.8926172	946.5536529	327.2388085	575.0574316	236.9268083	317.146121	329.6578736	720.890119	0.006993597	-2.00817

B2RZ37	Reep5	731.3891178	493.7385262	770.0346344	621.7382574	781.2463882	291.9801497	558.9504631	24.57574765	0.097552021	0.10510375	534.695256	0.00746884	-2.89121
D3ZC81	Pex10	29.39820599	26.87837026	25.60040564	25.51272206	38.26760534	33.23559597	0.10093384	0.104457878	0.097552021	0.10510375	16.90416869	0.007536739	-3.45789
A0A0H2UHT3;P0C2C0	Mrpl22	210.2385303	274.8446512	240.1528766	193.5979998	231.0775823	78.51054765	0.10093384	0.104457878	0.097552021	0.10510375	104.5337278	0.007959437	-7.52181
Q5XIU4	Bcap29	209.7588335	282.0885247	491.9860017	303.6245806	420.5197982	140.9902312	197.4797471	0.104457878	0.097552021	0.10510375	196.0704399	0.007966105	-3.83207
A0A0G2K6I0; P62246	LOC691716; Rps15a	1087.685024	718.8197758	1688.31	902.9911716	941.0785664	2237.332826	1282.489743	1850.509182	1575.199345	1607.766881	1540.550276	0.007998659	1.575524
Q68FS4	Lap3	1060.129181	704.3159437	929.2136922	960.0684396	849.3307948	841.4024387	711.8072604	591.523274	647.6656928	615.3722567	764.3977628	0.008199668	-1.29517
P08733	Myl2	55638.13717	63523.98811	39590.99478	55324.85426	54008.02495	73484.76822	59464.46609	76894.88795	75084.76918	73000.17089	56145.81108	0.008427497	1.287133
O35567	Atic	1405.494473	1756.198476	1284.60125	1354.999881	1406.029562	1778.513526	1648.240613	1669.508494	1868.514289	1829.580314	1501.210578	0.008721954	1.190406
Q496Z0	Elp2	76.31525153	63.52347767	80.93306605	69.81804731	83.7771564	127.532577	95.46149509	84.09352503	107.4386002	124.126172	101.7805437	0.008767329	1.425591
M0RC57	Smap1	195.461264	232.5900509	0.105148197	168.1653794	187.1901841	0.122760455	0.10093384	0.104457878	146.0601115	0.10510375	0.100763788	0.008813476	-6.41372
Q27W02	Magoh	250.792251	185.8347835	237.4329916	210.7762229	241.9137187	210.6117304	181.2632139	200.63855	183.9374344	158.7975635	150.5104043	0.009101622	-1.2453
Q4V882	Epn3	81.06136982	46.1861866	96.24988503	100.1696788	141.7605699	0.122760455	86.5386085	0.104457878	0.097552021	24.45211216	66.53944042	0.009220647	-3.14027
P15999	Atp5f1a	55084.75257	64760.72256	64752.54354	54161.27774	58546.7038	23500.26894	55448.46237	28927.19172	37600.01268	32635.03257	53831.42431	0.009301366	-1.53817
A0A0G2K5E4;G3V6I5	Dnaja3	1221.92123	1133.988061	1189.22226	1255.62523	1290.263904	1267.053338	1314.291172	1492.66508	1646.987606	1453.379839	1363.689778	0.009361322	1.168122
M0R7S5	Plin4	5781.049942	4344.164142	5738.298056	4630.360266	4901.920826	1260.314101	4511.45483	1129.496839	912.9326441	1247.190454	4577.264945	0.009440062	-2.23445
A0A0G2JZR1; D4A0F3	Osbpl6	104.4902728	116.0267308	54.31244285	89.39180885	87.42676459	222.7117535	163.5805557	107.2010211	163.1689879	146.3166535	131.6361057	0.009970921	1.724453
D3ZSW0;M0R7C8	Olr390;	32473.5743	37969.20037	40613.91423	35040.28951	28853.05339	26206.3937	27377.57273	14298.2168	9463.784473	5222.906077	27181.73839	0.010063133	-1.91288
D4AA35	Asmtl	892.5441985	860.4830397	1007.764735	663.7522283	1240.920312	1277.215243	1175.363602	1461.999833	1280.048752	1410.824624	1129.749768	0.010243847	1.381642
F1LT36;P63326	Rps10l1;Rps10	3063.545381	2646.083026	2141.994308	2131.834173	2814.585185	4039.248463	2770.974215	3355.258708	3497.598146	3915.451093	2938.662966	0.010542391	1.335959
G3V8C3;P31000	Vim	23722.13262	23812.56404	25763.64745	26989.97757	24350.2274	13327.97077	20231.9221	13422.62006	9723.32715	17901.9933	21652.44164	0.01055678	-1.55377
M0R757;P62630	LOC100360413; Eef1a1	5037.053116	7271.131907	6183.086225	5472.528084	5801.833704	4656.950242	5747.690333	4032.201353	3802.435826	3432.120106	4829.567039	0.010848217	-1.34783
F1MAA7	Lamc1	5421.327898	5463.109808	4975.888398	4822.507235	4799.616766	6137.11645	6664.862535	5408.599408	6278.014125	6618.02082	5168.187489	0.011051515	1.186267
Q6AYC4	Capg	741.4736005	731.303599	599.4162819	643.6823791	610.8405744	846.2139532	855.5031903	779.8374171	831.1438129	830.4109112	717.2410602	0.011168649	1.217504
F1LPC7;Q8VHK7	Hdgf	367.7118993	366.2028322	332.4117076	361.7437031	355.2434922	632.9227571	468.6899578	439.554403	480.5891018	520.9593042	383.6700548	0.01136282	1.367485
Q5BJT4	Txndc15	132.5181187	150.3743053	140.4811266	123.4215027	166.5698059	162.3833329	200.2405192	196.3996508	235.246722	194.1237203	146.7475681	0.011390458	1.326041
P15650	Acadl	31719.82009	35310.18171	26057.79337	30630.45315	27124.39739	44629.95675	33672.01399	36202.0566	40518.15564	38064.44392	30906.5781	0.011480051	1.237455
A0A0G2K9P4;Q5PPJ9	Sh3glb2	121.6171692	144.2436567	87.56752966	49.9112942	100.9745725	118.0779745	136.9464107	205.7624787	165.9705569	234.7725187	141.4301626	0.011544631	1.6573

A0A0G2JT00; G3V616;Q6MGD0	Cuta	594.6713619	543.590253	451.2736773	512.2122657	577.2667559	581.7332806	633.079666	687.0639789	750.5259749	707.1040179	593.0136515	0.011802619	1.22947
Q7M733	Hps6	859.9682102	504.3556247	509.9571083	821.8728924	721.3740754	225.9725595	558.6025174	104.3347095	0.097552021	0.10510375	658.609623	0.011860337	-2.64972
Q6P7Q4	Glo1	1732.333903	1737.200199	2127.448508	1875.739707	2405.412093	1856.618516	1488.079727	892.9696855	965.0513474	974.1182859	1798.717507	0.011986607	-1.48626
A0A0G2K4G3;Q71UE8	Nedd8	295.1171266	137.4529015	206.8715372	184.2631209	191.4956177	505.2037804	419.3158203	321.6496133	523.5388439	339.2820771	259.5364972	0.012060339	1.944219
P13471;Q6PDV6	Rps14; LOC100911847	1568.322339	787.2813579	1199.347009	1326.964374	1701.275208	959.9071883	1024.680664	519.4398787	512.5407228	821.9255027	1116.075069	0.012256271	-1.59445
Q3KR94;Q7TQ11	Vtn	814.280188	519.0692289	957.2633088	594.6402022	1295.24285	906.5316406	1918.944507	1884.797939	1969.193166	1853.341691	1179.130543	0.01226621	1.935962
Q9JJ54	Hnrnpd	564.5439955	627.8243894	535.9958874	540.2951338	536.1526053	488.4394931	1005.860914	882.330717	960.1930376	1059.186121	916.4921647	0.012391972	1.578389
F7FFR1	Rars2	71.58419779	70.33539803	88.57687117	89.00970199	90.32159522	146.7897618	111.2704718	109.8887283	112.2265771	119.6298578	83.06492616	0.012402511	1.388531
A0A0G2KAN1;F1LS40;P02466	NEWGENE_621351; Col1a2; Col1a2	1249.532925	1133.573847	2505.910524	1565.460861	1400.201783	253.4526117	1179.853014	302.436192	4.139009935	289.8884466	1135.796702	0.012442859	-2.97755
M0RAD5	Clpp	1136.933679	943.2800369	964.5661797	1050.582108	999.2012337	759.7264252	737.4149099	826.8754679	888.8903937	852.1268573	1036.52346	0.012721901	-1.19835
B2RZ72	Arpc4	384.2369179	497.0256865	356.6773302	400.1118928	411.4847353	587.7062766	486.0955931	526.5780994	629.8535774	514.8826408	402.5807008	0.012893269	1.279841
P07943	Akr1b1	12422.33463	10576.1468	11981.36188	12257.404	11781.57211	9980.175662	10938.79263	8015.670148	9746.147082	8632.000609	11600.81685	0.012987607	-1.20214
Q66H94	Fkbp9	85.65083009	83.69899253	93.43261897	47.78284103	99.23771676	142.981258	112.4341619	85.18336262	199.3440327	190.7145522	130.7194897	0.013066171	1.751608
Q5XJW2	Gadd45gip1	631.1644963	609.4099339	594.5927752	645.2611319	1001.96442	1297.903047	1011.790222	948.8319532	942.0919696	797.3144563	838.0437672	0.013388004	1.396543
D4A7D7	H6pd	92.4625053	141.3584923	92.5880368	53.76633483	97.90777218	228.121317	150.1069445	145.9651889	149.1080912	124.6041555	131.0562557	0.013521008	1.619248
G3V8L3	Lmna	1890.073051	2217.69876	2699.502577	2214.387165	2444.938342	1134.320019	2245.634523	1092.411742	1059.676808	1230.621216	2075.012257	0.013672584	-1.55696
P18484;Q66HM2	Ap2a2	279.817446	212.1387402	297.9509005	282.5929053	305.3936035	275.3851975	324.3541969	380.2246681	385.1144972	409.6298377	355.794882	0.013679289	1.288503
A0A0G2K327;Q8K4M9	Osbpl1a	323.8271504	199.2007515	373.2393586	312.6390926	297.8070954	433.4287003	341.1952829	435.11998	375.0902887	370.4906869	389.6250447	0.013885971	1.296945
Q9ER34	Aco2	37131.95986	49429.85703	53613.29549	47535.53386	52785.81039	37739.27813	38380.25432	30574.56794	26200.77516	27272.6454	45288.17046	0.013998984	-1.40466
F7EMK8;Q6AY99	LOC100910708; Akr1b10	314.2295034	459.6730114	270.1073896	230.9516419	222.0330256	430.9849175	508.3144062	358.6710817	677.83537	420.861101	396.3309043	0.014276686	1.554781
P62193	Psmc1	405.9375475	251.2864471	484.5740613	387.4691944	422.5364705	355.5236146	320.3222646	233.0311396	155.4903237	175.4382796	323.7099735	0.01431568	-1.49801
Q68FT7	Farsb	294.6637161	381.263122	232.2621731	171.8888718	219.9619135	620.6310144	339.2524728	322.2632651	499.6617603	411.3146153	348.5661073	0.014323077	1.629238
F1M609	Acox1	414.5269052	289.7303848	306.7250696	279.3670695	402.5872545	571.9975238	543.9573769	441.6313832	618.6994589	519.3580822	305.5194467	0.014370084	1.477296
D3ZE15	Ndufa13	15579.03154	14184.85468	13234.65651	13687.01214	15689.92834	17140.80861	14824.87164	19728.09848	21457.2167	20339.89942	17261.00421	0.0145979	1.2752
Q562C6	Lztfl1	55.15331816	58.33046256	65.99690711	61.65637806	68.87643408	0.122760455	39.33133127	0.104457878	47.37457834	0.10510375	52.11210734	0.014638684	-2.67348
D3ZZ20	Afg3l1	1051.317442	749.9644702	1817.572073	972.0172411	989.2128738	437.1841932	753.362321	363.8856874	969.7704502	164.0636886	745.6814314	0.0146537	-1.94997

A0A0U1RRP9 ;Q7TP05	Cfb	806.5267262	458.4034806	1007.106147	1068.433606	768.098245	1195.489852	904.3984403	1493.79828	1128.564349	1294.346909	1349.712123	0.01465472	1.494095
A0A0G2K6X6 ;O08679	Mark2	58.69140738	54.51844357	76.77564823	45.18535075	65.81436488	65.60718019	35.44961917	0.104457878	0.097552021	0.10510375	39.32424994	0.015189591	-2.56725
Q6AY23	Pycr2	50.51371174	39.23684417	93.53884855	47.8897824	15.08815112	0.122760455	29.0157087	0.104457878	0.097552021	0.10510375	42.82160411	0.015410177	-4.08928
G3V9S0;Q5E B81	Cyb5r1	341.6480013	283.6885372	235.4736567	280.951908	266.0510197	392.7944576	278.8564388	417.3228032	398.284139	326.1886607	322.7287504	0.015412468	1.264476
A0A0G2JWS2	Nebl	4094.924487	3733.365087	4260.344134	4280.714891	4647.990562	3768.455263	4011.549184	3814.454366	3410.992862	3616.13455	3966.402191	0.015552884	-1.11656
A0A0G2JW01	NA	422.9910094	250.1302257	329.8509023	421.5743606	431.7714406	395.2985941	547.7744306	503.2841009	510.7733069	511.4138129	407.575501	0.015579667	1.29114
F1LRU1	Ilf3	186.1502326	145.284237	171.8626233	149.3731664	186.543545	213.4946417	183.5219196	195.4145723	211.7541841	239.3169857	181.605295	0.015723073	1.216523
D3ZYS7	G3bp1	360.8062923	382.2115464	307.3896262	345.2473004	368.0961904	509.9417915	388.4063412	413.8320331	436.9939348	398.9156894	382.8644796	0.015840636	1.19582
P16409	Myl3	125013.8933	181428.8279	167477.4691	165985.0486	186588.7815	43409.42053	157755.1603	96821.76243	107143.9834	53963.67294	142017.9316	0.015960285	-1.64993
A0A096MKD 4	Ldb3	13367.87085	10700.93116	9559.219761	13413.88602	10802.37711	11189.91641	13511.25715	14967.64491	15319.87801	15388.69372	14136.28666	0.01601053	1.217546
F1M7P4;P21 807	Prph	545.8327215	478.3719062	400.747254	599.1076804	577.8353781	433.4297117	477.942709	321.2209815	159.5558447	304.6687898	414.008264	0.016315209	-1.47917
P32577	Csk	613.8468458	278.0230469	288.1907745	282.1415538	357.5334048	0.122760455	199.0040889	98.04321668	221.2862711	230.0400273	174.6664991	0.016446545	-2.36544
P24329	Tst	1351.292589	1349.04974	1439.105051	1256.355389	1493.684692	1003.453882	1250.055259	784.866684	940.9294906	1026.890389	1341.766274	0.016685912	-1.30237
A0A0U1RS25 ;F1LY19	Upf1	225.0688332	198.040949	167.4914066	168.0940221	196.8644581	237.3370239	203.7778394	254.4801182	259.5270771	251.2107446	225.8687287	0.016725127	1.249008
D4A9G1	Rpl3l	352.20322	390.0284624	367.4895478	436.2457443	400.8797161	382.1406199	289.9350737	249.9144374	222.2706629	192.1157858	369.6308375	0.016753408	-1.36941
Q3KRE2	Mettl7a	118.0856805	248.8793021	158.0411611	152.6481135	148.1407429	306.2427171	205.054159	214.534607	235.859161	221.5304662	203.8061087	0.01684689	1.399689
O08776	Ndufaf3	350.8306652	182.171611	391.994135	453.1415337	330.0321235	405.8703118	527.0887843	940.95955	1164.287417	593.7181513	866.954807	0.017764168	2.194785
G3V6H5	Slc25a11	7676.591082	7474.302529	6933.852054	6569.538416	6891.992164	8402.99273	8267.762391	7128.537645	8495.742618	8817.157697	7621.05116	0.01838085	1.142484
Q3MIE0	Echdc3	215.4695298	157.8078447	175.5137699	161.2054879	268.7380972	234.6347992	252.9624795	217.6240169	312.4709595	302.6639666	251.2716863	0.018388032	1.338146
A9UMV9	Ndufa7	7787.201112	6988.859284	7000.092337	6559.832561	7351.130584	5613.227815	6055.514397	934.7906442	974.453888	2932.988915	6863.233179	0.018428417	-1.83213
D4A3V2	Ndufa6	2009.636274	2169.907636	2196.716809	1901.326172	2342.561863	1896.570212	1485.4811	1108.662517	966.8879499	952.7178306	2171.121379	0.01845114	-1.48509
A0A0G2K531 ;P23764	Gpx3	723.7912871	750.144404	618.7993226	661.6663121	872.0886161	556.7741109	1129.130063	1307.903423	1053.374627	1186.893976	993.4075801	0.018518702	1.431017
B5DEH2	Erlin2	324.5938768	289.9567179	324.4176784	365.5651187	373.1362626	565.592049	399.6262673	420.4829983	471.2193385	399.7654555	356.3655221	0.018725481	1.297957
F1LSG8	Vps50	244.2380561	149.9911427	226.7702109	323.6830468	240.5686817	0.122760455	98.57551874	218.6759436	0.097552021	219.9526269	71.29726062	0.018771375	-2.33654
D3ZFY0	Sephs1	150.1725226	166.7639692	128.1942574	85.05515765	146.7333966	161.7211652	177.3064706	141.5259841	167.6053899	214.1930261	196.1631224	0.018890854	1.303104
B1WBY5	Dnajc11	362.5508642	270.5860574	286.4463695	320.1977643	303.1761093	328.639944	342.8371827	392.8579571	430.730814	426.9832789	318.6259656	0.018985538	1.210163
Q6P9U0	Serpinb6a	574.6608765	401.8669317	668.4827443	666.1762326	740.8451919	1028.957697	814.7050717	809.7131737	754.3776245	867.849375	604.5930615	0.019239538	1.332499
Q63488	Slc20a2	361.6967485	271.258894	353.6292262	280.6004505	396.7503606	541.0497359	390.1916269	450.5091871	387.6149822	459.3582041	364.6333927	0.019422174	1.298807
Q5RK09	Eif3g	233.141458	283.3851575	181.5493143	156.1989673	185.1439105	302.0114	274.4586645	264.5173733	319.5908756	348.7580938	239.9725687	0.019656596	1.402474

E9PSK7	Mapk8ip3	258.1037643	271.888496	33.73109034	270.5316603	277.5271082	283.0028418	374.3925301	405.9835065	394.3631837	401.7737429	261.4203754	0.019901841	1.589742
F7EUU4	Cops5	443.8493495	381.8472529	365.7271732	385.1391561	483.4377791	480.511763	437.9168723	530.9355052	613.9855957	593.9777033	426.3570248	0.019939779	1.247445
Q6J2U6	Rnf114	182.8415485	188.3366545	352.7971024	316.9218553	464.2762379	266.6972766	281.6020562	0.104457878	0.097552021	133.7717457	0.100763788	0.020019105	-2.64695
F1LPC8;Q924Z9	Ntn1	61.15889116	48.77841709	75.07992979	72.59378503	62.61722107	0.122760455	0.10093384	96.26455098	0.097552021	0.10510375	0.100763788	0.020074888	-3.97011
D3ZHV1	Tnip1	4019.181382	1360.188318	3478.747755	3462.881837	2586.395455	1590.254703	2284.43565	520.3491353	663.3473707	579.8975599	3205.653057	0.020436585	-2.02273
A0A0G2K7T6;F1LS02;P37199	Nup155	127.0672961	88.98212331	142.828157	126.5653628	115.9576328	247.1381546	194.1833183	168.1798456	143.7424025	134.4902444	140.1334684	0.020671148	1.424269
Q5U2U0	Clpx	156.6274687	260.7215309	191.5258624	154.3986063	189.5072535	337.5940619	266.3830948	212.9142316	297.0030934	340.1552582	196.142827	0.021045735	1.443313
P97924	Kalrn	107.8442168	109.5952604	171.3578202	131.917476	172.4725892	247.740212	147.0701044	211.0341141	200.3961375	297.5798678	199.7128565	0.021121089	1.567077
A0A0G2K7D7	Nars2	66.72848267	100.2766626	64.41746201	78.76273923	106.5377296	53.19873859	62.97999938	0.104457878	0.097552021	68.05632201	67.43005663	0.021133399	-1.98544
Q32Q06	Ap1m1	246.6207883	191.1797026	209.1292648	224.8233169	223.2698137	292.3450819	250.9886204	247.1615636	247.3442384	285.0944673	221.3685376	0.021229035	1.175244
Q7TP77	Mrpl49	191.456795	208.4952574	258.3041931	206.0072813	267.1552406	305.6962928	285.0882595	356.6072672	443.8807181	321.2176909	237.3786884	0.021683557	1.436153
B0K020	Cisd1	1658.431603	1960.242642	1673.327666	1951.238323	1787.257778	3305.173009	2042.086625	2865.232418	2310.707049	2223.778523	2027.206162	0.021787467	1.36336
O70595	Abcb6	87.82013277	158.9274702	109.9040449	89.20493343	151.6072788	169.1247431	165.06527	186.3184678	166.177849	166.076258	150.3013613	0.021939006	1.399058
Q5XI78	Ogdh	17756.89659	19847.89907	11993.29887	15888.37451	15939.3601	18872.50907	18158.3152	21361.8299	28181.80901	22944.2328	18925.80502	0.021941807	1.314535
A0A0G2JVC2;D4A1Y0	Scyl2	101.8074008	229.1211008	122.4967753	107.5435325	93.76212561	94.93669399	46.32340537	0.104457878	78.43440138	77.06887716	63.38773529	0.022399954	-2.18089
B2GUX7	Creg1	248.0141139	205.153584	270.6142624	266.868221	266.1242895	0.122760455	194.3994907	261.4247597	0.097552021	0.10510375	185.4420251	0.022443299	-2.35061
F1M6Z1;Q7TMA5	Apob	63.52732079	59.28172437	128.4565022	100.484228	128.4472382	136.5067567	226.3084052	166.8896149	121.8352934	162.3601149	136.608258	0.022629496	1.649511
P36972	Aprt	8926.560489	7587.683814	7673.020776	7732.166945	9083.155172	10249.67322	10198.71891	10533.79358	11146.48729	11422.51133	8810.724631	0.022810232	1.267438
Q5RKI9	Mrrf	363.4051789	236.5721767	352.7834006	345.7606756	331.195547	503.1784652	360.2680762	438.1544163	457.6713133	411.9210597	359.8967751	0.02284957	1.294238
D4A3T4	Zfyve1	10.13630175	0.077969763	18.00522689	24.52529601	0.103353746	37.0244946	14.81885725	17.51670601	32.73264684	15.93419143	29.05144931	0.022983279	2.319197
G3V6R5	Suox	392.7564437	359.4237363	374.0943937	431.5212159	461.4686736	410.7528197	393.56038	256.6730547	255.511511	297.2043644	309.0436577	0.02307804	-1.26024
F1LPV0	Nars	343.1248733	212.7859739	331.3328218	317.4036038	310.8667659	398.8954663	368.6515483	353.7002154	369.5377603	433.6817506	322.9041216	0.023237356	1.235758
D3ZLA3	Cpne3	154.6866685	127.555729	197.4020705	166.2306126	204.5568653	252.3430735	207.490919	241.6018466	208.8240659	278.6977904	170.3730139	0.023317692	1.332
Q6P7P5	Bzw1	25.33430701	0.077969763	28.68308321	30.47783506	0.103353746	30.68801354	25.07184666	44.242038	44.4432656	33.17841292	37.78033578	0.023758351	2.11987
P11608	Mt-atp8	1674.576025	1672.54659	1710.019279	1533.301576	1048.170811	5122.988012	2050.881742	2967.340249	3425.504916	1428.327759	3027.684182	0.023824405	1.966186
F1LQH9	Bag2	77.76428017	131.3492834	56.61881295	64.07234551	62.93064853	136.9475805	149.4262233	66.65403553	150.8178415	140.9446644	94.69263006	0.023834712	1.569087
A0A0G2JZI0;M0RBF0;O88453	Safb	67.02508193	121.3014918	78.39650378	88.91818775	108.0603191	108.85012	130.4367818	142.8353736	114.0244557	120.4098606	102.0829995	0.023847343	1.291491
P04256;Q5I0M7;Q6P6G9	Hnrnpa1	656.5475333	369.5684191	569.7492674	584.5735562	481.2921353	805.2467722	507.4763609	741.5271844	701.956036	856.3682145	658.4908639	0.024087889	1.337183

F1LXA0	Ndufa12	1943.047773	1859.725036	1921.022907	2130.180626	2216.121827	1482.368763	1560.501852	613.6112316	724.7430426	1024.746937	2343.50913	0.02426063	-1.55935
G3V6E1	Myh2	25838.04948	37538.95957	76859.12623	58090.65259	57870.62092	31244.32215	1568.983153	6833.521157	14668.76495	17303.99607	1207.570483	0.024410829	-4.22146
Q9Z1W6	Mtdh	194.5912969	193.7411788	182.2798453	215.2817131	180.4715282	111.1195944	0.10093384	0.104457878	0.097552021	193.0998825	152.246341	0.024500904	-2.53879
P01048	Map1	909.6654717	966.9184285	1907.147504	1653.711843	1210.014489	1755.639201	2340.594145	2251.275374	2050.162177	1901.077166	1235.927519	0.02472812	1.446001
B2RZ76	Dhrsx	89.24991457	82.46501062	91.78603632	71.2906707	93.09699562	135.7765084	97.19492475	134.1807476	111.3451913	113.0962122	75.06436349	0.025232217	1.298348
D3ZJW6	rCG_21066	282.5206497	184.2957445	242.6418192	208.6739256	0.103353746	0.122760455	221.9076738	0.104457878	0.097552021	0.10510375	83.89699311	0.025480256	-3.59817
D3ZD23	Abce1	346.3007012	153.3131977	292.4529859	267.5014185	271.9271507	434.3579454	281.8237114	354.4972295	374.1906834	394.0880954	298.5329873	0.025636823	1.337776
Q7TSE9	Hax1	66.07317498	91.71422372	57.7077023	65.92538093	56.12838913	85.47301601	76.24066467	76.31378324	99.58941809	82.39896007	85.13725979	0.025715927	1.247111
B5DFC3	Sec23a	440.5050041	346.7577901	495.0133444	389.1435213	531.4915306	473.6252942	508.5489678	511.9828779	572.4157764	622.1664084	485.4798578	0.025730995	1.200767
Q4V8F9	Hsdl2	10486.71819	6974.14173	11989.42358	10429.79827	10376.07319	6870.40517	7721.258381	8615.152279	8374.546616	8777.562118	6602.514749	0.025779709	-1.28419
A0A0G2K309	Slc25a15	213.2525324	139.698256	205.3541756	83.51095623	72.95784728	184.8686367	242.6927971	347.5846415	331.8689204	350.888876	250.033157	0.026196059	1.991233
Q52KJ9	Tmx1	359.2979497	577.490634	794.1689823	615.7254505	596.4447929	0.122760455	393.4997298	268.8868769	0.097552021	549.3617259	509.1912979	0.026432298	-2.05196
B0BNB9	Htra2	290.2574486	299.7571183	247.4149467	256.9160051	244.4760355	286.4195334	310.5721136	331.7697353	381.7800507	324.5537278	279.6208346	0.026605479	1.191792
P08082	Cltb	254.0311033	0.077969763	408.8474323	226.300678	335.8919794	40.56610124	209.7899453	0.104457878	0.097552021	0.10510375	188.9093936	0.026735828	-3.34457
D3Z865	Mpdu1	47.13889353	33.71969764	51.79076973	29.4215605	39.79562383	0.122760455	30.28849179	30.00776931	30.69595851	28.64170207	29.64472771	0.026971963	-1.6214
B4F763;D3ZPE5	Vps53	58.68864542	43.30549558	80.45382156	86.45766138	50.68562435	0.122760455	0.10093384	0.104457878	59.22653262	70.93370876	0.100763788	0.02703788	-2.93676
F1MA54	Pdk1	4430.679213	3596.729549	4211.873793	3761.916815	4788.181239	5856.982845	4733.927206	6424.278857	6269.14394	5638.442066	3406.84459	0.027078487	1.295919
P40329	Rars1	201.9140072	220.397948	223.58496	206.2460429	240.8530243	338.5557504	239.6410176	309.8849046	271.6737743	256.8674484	226.7379579	0.027097489	1.252948
Q6IRS6;Q9QX79	Fetub	3901.062632	1664.505388	5491.429804	3972.692486	4100.452139	5732.310251	6168.231572	5643.027267	4378.785835	4669.576402	5205.487803	0.027174533	1.385136
D4A2G9	Ranbp1	322.1114652	255.7204064	362.1830744	252.4137785	297.6845267	0.122760455	306.331309	62.61354101	53.05775833	0.10510375	298.2421781	0.027921016	-2.48189
A0A0G2K6G2;Q7TP48	Apmap	194.2181864	207.202408	182.0700816	194.0060262	222.6588792	320.6942316	228.7855783	242.7398758	228.7015554	265.6058976	213.0693629	0.027953802	1.249469
B2GV57	Cars2	539.2758101	427.366147	405.9827472	445.5897991	461.9555413	550.3171239	502.3327024	454.5787345	594.8870963	562.0174132	484.8701643	0.028103782	1.150866
Q5PQN8	Ppp2r3a	174.7622155	158.2448185	103.289011	164.4357383	161.808604	274.4262063	161.2663745	209.0230258	186.611928	178.2336102	181.4748969	0.028494904	1.30161
Q9ESS6	Bcam	269.7043746	364.499861	292.0357368	223.3938847	379.1060706	313.1961708	376.7947604	351.1235155	365.0123148	428.2492518	463.9180738	0.028657611	1.252826
D3ZYM7	Lyrm4	454.7937121	351.4262439	651.9447504	473.2208742	645.9728998	433.8245024	500.7766802	90.96921429	76.38921915	228.9156501	402.9083492	0.028961707	-1.78386
A0A0G2JTG7	Hnrnph1	750.5651318	827.6630232	653.6146455	829.8839561	728.9144704	791.5501851	887.9892371	912.7969818	852.6393744	863.6972508	763.4266568	0.028980866	1.115049
Q05BA4;Q6VBQ5	Myadm	253.8730208	333.3964263	266.9510986	339.9110793	343.8799737	296.8499201	282.5648211	188.3432477	131.2167564	251.6685367	263.2210115	0.028993233	-1.30537
B2GV06	Oxct1	6696.487146	5268.545554	7040.606226	6802.138891	5882.330022	7035.486214	4572.985538	4031.387061	3813.492833	3423.957886	4994.211035	0.029274306	-1.36441
D3ZMY8	Pcnt	568.9695761	24.6702199	454.031828	493.2584346	383.2162493	684.8939474	535.5485235	907.9267863	474.2302688	659.5356541	625.2054801	0.029309074	1.683578

F1LWG8	Srl	11631.27243	6846.831727	8298.819925	8888.918382	9630.183134	12989.5691	9375.980963	12073.48536	11788.61013	11611.47433	10877.35548	0.029693872	1.264211
Q7TQ77	NA	115.671668	135.6942512	128.8305439	103.9695894	109.9348279	165.7927003	146.7370209	174.5136883	182.0496265	170.508863	100.7328377	0.02969556	1.318989
B2GV54	Nceh1	1438.899699	1064.42489	896.2123428	1070.081603	1094.914474	1288.99587	1289.356373	1589.6139	1702.108665	1487.588467	1161.503523	0.029707579	1.275813
E9PU01	Chd4	158.0364136	73.82220592	158.616463	151.1075462	178.1840759	230.2416958	161.3104376	219.2178539	163.608996	253.0676533	177.5404257	0.029947729	1.395113
Q498E0	Txndc12	183.9664948	232.9601265	135.6671021	153.1823474	162.6918799	354.7801886	198.9601798	246.9933593	255.1308057	207.2677513	185.1711732	0.030072896	1.389711
Q5XI73	Arhgdia	2381.790648	2655.069346	1964.500249	2101.646028	2354.852027	2065.94952	3419.037234	2901.147209	2936.495878	3606.671364	3078.220075	0.030178005	1.309692
Q3B7D0	Cpox	392.8115009	325.180096	374.8505314	331.107013	412.0467655	670.6963231	424.604528	463.6899811	485.4679234	517.5298004	389.5175403	0.030398097	1.339648
B2RZD0	Mrpl57	85.3886126	0.077969763	84.24346297	54.52353265	106.4692066	0.122760455	0.10093384	0.104457878	0.097552021	0.10510375	79.85428738	0.030535106	-4.93678
G3V6B8	Tsr2	47.29230923	9.603260125	42.41991557	31.6760655	19.47043844	0.122760455	0.10093384	0.104457878	0.097552021	29.88761596	31.15564447	0.03075768	-2.93733
G3V9G5	Synm	0.108550851	0.077969763	0.105148197	0.103324622	0.103353746	0.122760455	34.29460005	0.104457878	52.86957147	62.01415943	43.3427818	0.031401837	322.3127
D4ACG2	Ilvbl	328.164646	304.4224789	351.5762565	354.2435868	372.4781583	450.4006853	391.6138429	424.9722835	434.4793981	432.1991744	317.0163834	0.031655399	1.193671
P60841	Ensa	29.47819135	0.077969763	0.105148197	0.103324622	0.103353746	0.122760455	262.0705203	553.6694069	810.8558028	589.2742078	97.23428806	0.031674688	64.54031
A0A0G2JU01;<br>A0A0G2K446<br>;A0A0G2K4L2<br>;E9PTJ8;Q9Z1<br>I6	Arhgef1	127.3201635	151.9779583	134.6477088	114.215078	150.5067797	260.8925854	149.6786041	176.9541097	211.2973407	206.1724982	130.5270366	0.031734809	1.394303
F1M779;P11<br>442	Cltc	4047.248243	3089.333323	3088.313194	3693.818927	2535.256546	3063.20304	2417.26345	2729.986498	2324.601066	2866.886713	1861.279371	0.031951289	-1.29362
Q5U2V4	Plbd1	310.0217997	218.4085627	211.1457177	253.3374978	226.1787826	617.8123717	293.5171038	370.4589021	409.0471028	339.5632476	283.254149	0.032360178	1.581541
Q9JJM7	Gp1bb	0.108550851	409.4539931	256.2207552	0.103324622	240.1340143	0.122760455	0.10093384	0.104457878	0.097552021	0.10510375	0.100763788	0.032389613	-1721.46
P70582	Nup54	112.0603183	84.22639131	90.86874922	79.86072889	129.7673025	0.122760455	105.9606105	0.104457878	0.097552021	72.52310309	86.61409906	0.032440077	-2.246
D3ZS58	Ndufa2	3704.857764	2559.50518	2512.544668	2718.210739	3773.144248	2916.763842	4186.603109	3360.416826	4740.722886	4406.72619	4177.124463	0.032559479	1.298355
F1M790;Q62<br>786	Ptgfrn	149.9722196	133.9265472	171.9845925	119.6523202	159.9515137	105.967115	126.3827485	123.4458023	93.26917512	145.003434	108.4170836	0.032788264	-1.25637
A0A0G2JYK0;<br>F1LM05	LOC299282	2358.646473	1818.867434	2551.327563	2769.60036	2067.968042	3524.166751	5742.186778	3324.761904	2569.326839	3959.262884	3140.567669	0.033102789	1.603802
G3V936	Cs	36146.63616	37052.28206	28383.76596	31660.79337	31760.81213	23318.87837	33068.39611	17853.22035	22016.40317	22876.0488	33684.9211	0.033139821	-1.29569
A0A0G2K527<br>;Q9Z272	Git1	108.8357603	115.3162751	139.7185069	121.9010135	158.3114417	273.2387814	937.9549994	522.3710303	241.3561972	844.4966356	1568.009146	0.033245893	5.676581
A0A0G2K1B0<br>;D3ZT82	Rbfa	124.9332076	71.49905534	168.4275137	146.5544267	136.4321494	109.705384	104.4253183	74.09495388	82.24318887	100.3477476	90.97994818	0.033423805	-1.3838
A0A0G2K0I6;<br>F1M7E5	Cdh5	196.4775865	212.9037565	149.9145577	241.7143449	186.9913965	229.3406609	327.6531207	287.5014378	397.6084377	298.5391384	175.5834379	0.033546786	1.447557
F1LTR1	Wdr26	126.5508365	141.81123	90.34145995	123.6442223	156.7977073	169.6316994	118.0278612	233.5415704	231.1580396	147.3232028	193.4274508	0.033998901	1.425223
A0A0H2UHM<br>7;Q6AYZ1	LOC1009094<br>41;<br>Tuba1c	477.5565287	277.2122023	488.9328918	98.87509393	508.6508825	257.2276981	878.784199	572.9023057	845.3482777	1065.476308	714.0377017	0.034033953	1.950857

D3ZK97;P84245	H3f3c; H3-3b	6928.919535	5855.426015	5726.518172	5779.489999	7152.880132	3476.353413	6855.736903	1944.468355	1486.221828	2121.793887	5176.065565	0.034415548	-1.79158
Q5I0H4	Tmco1	224.753513	214.7782146	267.9912853	466.2022856	247.8024346	575.0633508	424.9366413	569.7125915	532.3223364	539.4652686	179.0941802	0.034640523	1.653499
Q7TSU1	Arfgef2	60.80487436	48.07171498	46.71071803	76.79137147	68.60246055	0.122760455	51.79250448	0.104457878	0.097552021	0.10510375	69.70793542	0.034683649	-2.96216
E9PTY6	Fry	87.40432153	125.8607051	89.72353621	171.0490596	214.426757	213.9760292	219.0007589	235.6138963	206.686677	249.4050276	169.4313363	0.03471315	1.566425
P56558	Ogt	0.108550851	38.1157195	0.105148197	76.3246559	0.103353746	135.3051049	39.8231635	63.42638655	63.92082917	47.94890299	47.8551002	0.034989414	2.892184
P05505	Mtco3	1874.395721	1103.34248	2967.498532	1749.902753	4038.302877	1248.575133	2201.96117	437.0267315	339.9130027	248.8393348	1352.659022	0.035117657	-2.41554
P62632	Eef1a2	17248.84575	13533.15538	19296.83001	16529.19099	19228.1308	17352.15799	14461.43294	11808.3327	11734.68215	13427.10568	16780.34322	0.035580016	-1.20382
Q63083	Nucb1	316.8541296	320.0932162	288.0541409	256.6889104	301.2813116	319.7656608	292.5815375	104.8839438	160.8302609	135.7726798	243.269317	0.035691751	-1.41561
G3V8B0	Myh7	117628.1359	88354.08646	133341.4273	129204.7281	135882.6262	125102.2453	108524.4275	81702.41489	72512.5053	69758.55776	108438.7298	0.035979725	-1.28135
Q9QX80;Q9QX81	Hnrnpab	739.2717754	561.7858943	541.2308352	578.4780719	600.344088	849.8462661	705.7490737	619.7634334	883.4683494	936.7315271	670.5178191	0.036144514	1.287075
F1LMQ3	Psmd8	285.9266447	295.1213106	335.4233267	277.1325737	283.9369771	449.905605	351.3274165	270.5764754	388.7635584	487.1741643	360.3461039	0.036190989	1.301765
Q5EB77	Rab18	412.3094109	264.7270704	298.9095178	341.3308784	270.6205939	115.5657366	246.24769	292.3112564	246.3232289	276.6708995	247.493321	0.03645986	-1.33754
A0A0G2K8N6;D3ZC34	Ankmy2	169.807529	94.19593484	135.8333856	126.563794	153.3075786	365.597592	142.6729923	275.9383256	254.074667	312.8113475	128.6025676	0.037033858	1.814133
P54001	P4ha1	109.7893319	41.73975909	118.0011909	154.3146993	190.0721538	156.9786023	210.0775335	258.4700342	202.59405	281.7175988	88.54499498	0.037157562	1.626689
F1LND1	Cog1	59.80870962	52.81022572	76.25009176	66.41420449	71.57537107	90.86686346	61.94030891	80.95513449	78.3395674	86.65573732	75.04469075	0.037186667	1.20797
D4AAI5	Cand2	1063.565786	846.4760837	938.7286928	977.8351161	957.2851202	1147.369462	1016.456564	1196.127373	1175.06994	1127.037057	1024.530269	0.037324363	1.164776
A0A0G2K2C7;D3ZC84	Usp9x	256.0957491	263.1190392	216.0134747	198.6626343	246.5842558	252.9135304	284.0278504	360.2744821	290.1150002	288.0013392	244.3260944	0.037364793	1.213959
A0A0G2JXC3;P05765	Rps21	433.7480052	233.1414405	170.1896798	271.6627508	200.4250968	317.4286866	377.7305422	469.9175106	771.2564343	460.9814296	307.5825705	0.037411273	1.721767
D4A3E2	Npepl1	243.9625712	173.1769394	212.7770261	204.6590602	243.2206225	188.2709984	159.4942875	147.9029099	236.6920954	152.7661741	178.3228458	0.037433615	-1.21619
D3Z9I1	Coa3	234.4427564	189.4425626	0.105148197	0.103324622	0.103353746	0.122760455	378.1350415	400.0623566	472.4041752	325.2589634	427.9788458	0.037954087	3.936774
A1L1K8	Habp4	137.2824238	107.5268893	71.47702848	81.75300609	102.7611055	141.8088372	118.8452303	219.164251	287.6921865	125.796115	211.7230372	0.038136764	1.838772
Q60587	Hadhb	62278.74395	56171.5613	53017.53238	58697.53138	59095.83517	75285.94702	62392.25715	81370.10827	70636.48086	80319.46467	50956.75152	0.038157113	1.212748
Q4L1J4	Magi1	82.15445614	0.077969763	79.5838011	37.96321016	80.50256352	126.464567	101.033933	60.66411277	80.38799462	112.3101553	92.30071242	0.0384419	1.704121
A0A0G2JSR0;Q9R1Z0	Vdac3	7642.80463	5892.084367	4629.639698	6470.779652	5665.117085	6538.670815	6871.729101	6898.662987	9466.252806	7252.69505	7168.199606	0.038641384	1.2155
A0A0G2K7C6;Q99ND8	Ppm1b	328.5861882	451.863662	401.2019184	309.1042415	380.7770214	466.5881844	466.4498721	426.0401969	439.4268408	387.9805436	417.0288713	0.038895063	1.159261
F1LZJ4	Hyi	277.1956023	157.702221	334.4827753	265.0745764	278.9576846	539.6447791	345.9021302	345.2358375	314.8134226	408.6241223	334.8425763	0.039050291	1.452363
D4ACP2	Smim12	488.310808	552.3727967	531.8309689	481.8776537	440.8471284	732.1771777	733.5959464	1156.41336	1329.277036	1178.358659	429.4839373	0.039159948	1.856638
A0A0H2UHM3;P06866	Hp	357.7822858	259.85653	492.9770286	482.7931588	279.1681581	333.2065221	1154.134475	518.4333304	758.5768151	1064.114527	1144.121646	0.039295826	2.212898

D3ZM91	Sync	120.7984994	145.5298675	67.63243521	131.2981842	114.3171823	194.5470367	151.4358441	151.3672054	313.1873274	220.0334241	130.9909412	0.039456979	1.670131
A0A0G2K9B1; A0A0G2KB85; A0A0H2UHI5; P09006	LOC299282; LOC299282; Serpina3n; Serpina3n	1450.478942	950.5765673	1729.931461	2040.337899	1136.422887	2373.046068	4216.009455	2728.282172	1896.607175	2613.374466	1908.452864	0.039877328	1.794417
D3ZN76	Sec16a	239.7463042	71.78344105	388.4197445	410.2435898	315.0933311	147.4161893	132.6548897	72.65449001	55.61467337	330.1292174	134.8706762	0.03997071	-1.95839
P02454	Col1a1	484.3041527	254.8733466	646.9043242	388.7228821	662.0996547	430.6824371	337.8913383	230.0434683	289.7963962	263.96726	340.8896643	0.0399998	-1.54457
Q5M7T9	Thnsl2	368.9161353	287.6434896	411.0295591	425.0869185	460.0450993	464.6429662	448.7701864	522.360893	438.6187202	496.5543725	421.0581313	0.040236454	1.191502
Q68FU3	Etfb	6957.283215	10044.77754	6930.828017	6383.38256	8309.48072	7036.091024	9962.660879	10047.1892	11321.46075	10921.93009	8679.234776	0.040324687	1.250646
D3ZF13	Ndufab1	6821.337585	4836.287356	5794.406017	4836.404094	4903.328314	6346.284561	4283.773122	3288.704553	2830.460929	2005.720574	4644.196602	0.040608392	-1.3945
A0A0G2K850; A0A140UHY3; B1WC50	LOC100912481;Ewsr1;Ewsr1	701.404993	533.9730938	855.5901876	638.8716416	836.832145	765.9404239	492.5775305	279.2520452	260.2453204	196.7991701	299.1982472	0.040691288	-1.86573
G3V8U9	Psmb4	817.5048453	728.7687727	1023.322484	822.9495269	940.1455818	257.1238304	843.3363009	570.1836849	816.3828179	458.8843905	784.7560579	0.040741525	-1.39365
M0R4H5; P36202	Pdlim4	227.3914895	297.5955799	261.7144822	245.3039043	289.7310069	256.1967021	141.7104738	200.0880805	173.2416924	0.10510375	213.835931	0.04089731	-1.60995
Q5BJQ2	Mindy1	80.75098554	27.37522584	76.40677277	71.82139658	0.103353746	124.134664	59.05440518	89.18237188	142.9091259	121.3240817	72.33965444	0.040998505	1.978703
P60825	Cirbp	172.5158508	127.657498	212.7835883	173.6385605	200.2134247	192.7386915	269.1423268	216.7295359	199.262774	241.6388555	180.3960558	0.04100145	1.221522
A0A0G2K865; F1MAB9	Tpd52	164.7235144	189.9886222	102.5430213	208.5439816	275.9987862	342.7216561	281.7218782	313.7354499	215.0756953	305.3855404	226.8564249	0.041008388	1.491382
D3ZUC9	Oxsr1	357.5322094	268.9620125	327.4578566	300.2587418	361.1740947	398.9705433	362.2902337	425.9576329	319.2865317	404.7797268	336.7150459	0.042192779	1.159682
Q6AXU4	Rnf181	326.2206489	0.077969763	0.105148197	265.4939858	349.514658	871.0806629	331.0438977	367.7068412	401.1643768	366.3962979	235.2861823	0.042281265	2.277321
P55314	C8b	271.2559315	283.1110926	303.0885233	242.9412302	287.1471632	244.4107542	263.0307286	399.6396223	402.1298345	433.0830685	380.1532855	0.042311382	1.274703
A0A0G2K8K0	Sfpq	1021.743033	821.0091343	1081.717855	1052.373829	1022.898726	822.9427399	884.1894328	882.8442081	894.3342299	1023.126822	866.9526545	0.042667628	-1.11635
A0A0G2K6J2; F1LPA4; O08874	Pkn2	158.6321216	33.1104845	0.105148197	97.78709997	163.2360735	0.122760455	62.34368003	21.93952152	0.097552021	0.10510375	53.50295235	0.04304045	-3.93483
B2RZD4; D4A4W9; F1LQ14; P11250	Rpl34; Rpl34; Rpl34	371.7978278	307.2148328	510.0455447	435.5091843	580.5439351	507.0736594	311.2412376	215.6247714	162.2390314	161.5007904	341.7729158	0.043542401	-1.55705
G3V8Q8	Sec23ip	79.83813543	40.05193906	39.2444275	33.04122725	56.98357768	68.63371263	60.04787256	70.2358094	81.50389769	61.51075647	63.95223299	0.043907916	1.357513
Q5M7U6	Actr2	602.8991374	497.1151537	618.1484357	629.2684555	573.8094772	804.4712025	615.6537934	857.3406488	682.2283071	789.7049549	587.7956322	0.043922913	1.237258

D3ZQ74; Q63321	Plod1	120.4924879	149.728059	145.2899527	90.96541404	119.0399435	0.122760455	75.82449124	108.1270695	109.8330724	103.6176537	74.93703267	0.043977111	-1.58874
B0K036	Sdhaf1	201.9965764	192.6879397	253.8784418	187.0780926	227.7463417	255.0556383	194.4495757	0.104457878	0.097552021	0.10510375	157.8408253	0.044250334	-2.09999
D3ZUI1	Apip	174.9192876	250.5826777	142.3314723	135.3538679	184.8664123	156.8047154	258.2936948	293.0465547	502.9461625	391.8618255	226.6430614	0.044497116	1.716859
B5DEL8	Ndufs5	2651.283241	2260.636381	2374.582326	2601.572476	2903.528703	2579.166597	2373.30696	1187.295122	1175.32561	1053.405927	2427.894129	0.044672626	-1.42176
P05065	Aldoa	25505.63371	20321.29431	20691.52227	22046.42715	20057.75657	11636.40602	19881.522	13038.53946	15898.55869	14833.82187	23758.16668	0.044907073	-1.31601
A0A0G2JTN4	Pfas	58.658595	104.3696944	70.18541887	128.8109659	135.3961517	131.5882276	131.7994563	183.8929015	202.9107636	164.1048088	118.2077349	0.045340741	1.562232
Q9Z327	Synpo	1888.086751	1776.250944	1463.690117	1726.369788	2060.092366	2683.947561	1833.309931	2456.526433	2520.726832	2318.139418	1559.198711	0.045721817	1.250011
Q8BHI5	Dgat1	70.56357697	65.05028638	77.1812171	64.77781086	72.77351344	216.6881257	79.25990885	167.9021084	124.3012297	95.34531206	98.18409931	0.045847203	1.859305
Q5FVM4	Nono	494.3767428	539.8532818	489.1286328	526.3445439	535.0595815	280.4185522	872.237607	1398.509156	1460.054975	858.5239832	998.1632938	0.046083033	1.891827
F1LMM8; Q64536	Pdk2	2102.97225	535.8179277	1696.51559	1988.023628	1292.086599	241.9847292	1248.395001	719.2827695	665.1127622	929.6889857	936.3724099	0.046132167	-1.92761
Q8R560	Ankrd1	621.610047	543.4952654	518.9233602	580.3977505	672.2411779	680.3728499	437.8990684	379.2779229	306.3531783	337.7229057	425.9776232	0.046139343	-1.37249
A0A0G2K5T1 ; D3Z8E6	Camsap1	67.92221324	42.21117837	0.105148197	139.659523	100.5632692	74.32197182	0.10093384	0.104457878	0.097552021	0.10510375	44.21590473	0.046559585	-3.53567
M0R6J0	Mrpl39	144.6296812	312.8365889	152.8232364	186.1543419	264.4347611	334.7957245	248.9877783	297.3446673	473.027927	554.8332279	199.0250256	0.046874368	1.655871
P19945	Rplp0	2144.838134	2016.406189	2344.586338	2121.057737	2401.737687	2457.421552	2168.589673	2474.949376	2467.07465	2733.023385	2295.198109	0.046901257	1.102907
Q66HA6	Arl8b	587.3480885	539.7438271	681.3381768	504.9321493	641.1637712	1188.304424	599.2451445	947.6594161	633.8200816	940.3023859	869.9111761	0.046921881	1.460822
Q5XIP9	Tmem43	136.167134	110.176048	191.0495862	126.6813144	154.5280757	159.7950721	139.1901772	203.2094322	158.6299222	205.6180959	196.2704115	0.047061768	1.232385
P62078	Timm8b	0.108550851	171.2102896	0.105148197	40.98885217	155.3122968	0.122760455	146.7770796	340.4083266	496.3586026	330.0734067	184.1879821	0.047496545	3.394583
A9UMV7	Uqcr11	1382.680641	939.8370707	2950.791109	1458.742746	1843.60727	4411.55341	2885.575769	4412.651686	3376.13421	2667.703046	3780.042812	0.047621717	2.092518
D4A2P1	Ccar1	98.26144393	55.49722964	78.61491539	93.28310753	75.13403062	86.31360369	47.26196718	55.00235821	47.79469984	65.1692205	52.12451138	0.048000094	-1.35989
E9PSU5	NA	427.9446688	533.3495357	790.9990055	734.5819381	453.0726956	463.9137388	576.0488429	367.5036508	328.1407291	376.8995202	403.1385487	0.04807613	-1.4024
B0K017	Adprhl2	572.3555623	629.6715551	994.9463331	356.6313921	855.6403316	1335.24758	692.4945458	1392.537002	1290.651818	1363.190121	560.9949491	0.048123836	1.621844
A0A0G2JZS9	NA	404.5186811	124.4263024	425.6304554	122.5055246	152.1488546	88.63549887	0.10093384	0.104457878	0.097552021	274.5169591	169.0065773	0.048731936	-2.77029
B5DEZ6	Gnpda2	108.1488536	113.9698762	145.4751846	117.1363761	131.6218301	180.3074907	129.125532	209.2446895	176.7575455	162.1373692	110.1042882	0.048734698	1.308339
D4ACM1	Elp3	133.1760393	0.077969763	152.9652769	110.3610031	82.00563698	246.8739431	151.5585616	134.2542584	151.4292237	159.1981015	134.98656	0.049075474	1.703457
A0A0G2K1A0 ; B5DFB7; D4A5Y6; M0RBI3	LOC102553099; Naa50; Naa50; LOC102553099	263.8436954	336.1324588	525.5363886	284.1205445	267.875323	297.0072294	335.5989499	0.104457878	0.097552021	0.10510375	231.0817203	0.049161089	-2.32989
Q66H98	Cavin2	2631.819088	1786.955034	2958.375077	2757.163566	3134.500103	2248.370926	2359.885913	1974.978676	1751.279314	2062.958752	2545.456146	0.049290775	-1.23021

Q4FZS2	Bub3	47.81923287	69.37650415	93.14029106	82.07648675	109.0656982	197.104465	77.4353498	144.4730907	122.8141404	124.4636023	100.0880577	0.049299422	1.590744
F1LQZ0	Tmem65	2724.119908	1794.690906	2029.952306	2513.726367	2554.033065	2750.015223	2215.190749	3071.268187	3138.730315	3301.846881	2671.21383	0.049459151	1.230163
Q9EST6	Anp32b	311.7993993	407.4046218	199.5252503	352.0918629	218.763432	261.3949899	269.5031481	180.0227353	172.8677301	218.6464319	217.5287172	0.049609472	-1.3542
A0A0G2K9N2;D3ZYG0	Vav2	110.7245595	0.077969763	0.105148197	146.2434406	0.103353746	328.2171682	0.10093384	156.9961296	158.2015691	174.7338778	134.707267	0.049836361	3.086946
D4A2Y7	Naalad2	0.108550851	9.998457493	0.105148197	95.36919723	0.103353746	0.122760455	96.26272367	160.4017217	112.5510608	207.0517735	84.92534926	0.049942751	5.214531
