# Supplementary figures and images for: Cardiovascular Protective Effects of NP-6A4, a Drug with the FDA Designation for Pediatric Cardiomyopathy, in Female Rats with Obesity and Pre-Diabetes
Source: Cells. 2023 May 12;12(10):1373. doi: 10.3390/cells12101373 (PMC10216951; doi:10.3390/cells12101373)

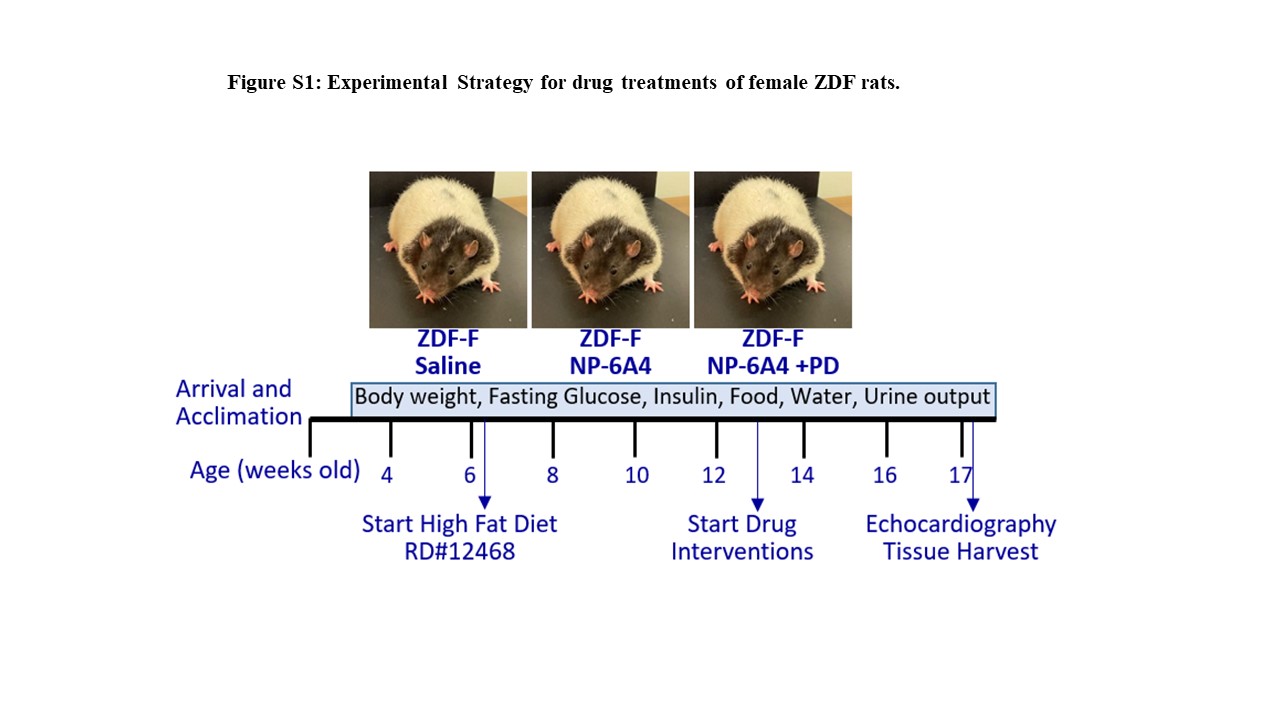

Supplement: Supplementary file 1 [file cells-12-01373-s001.zip › Figure S1.JPG]

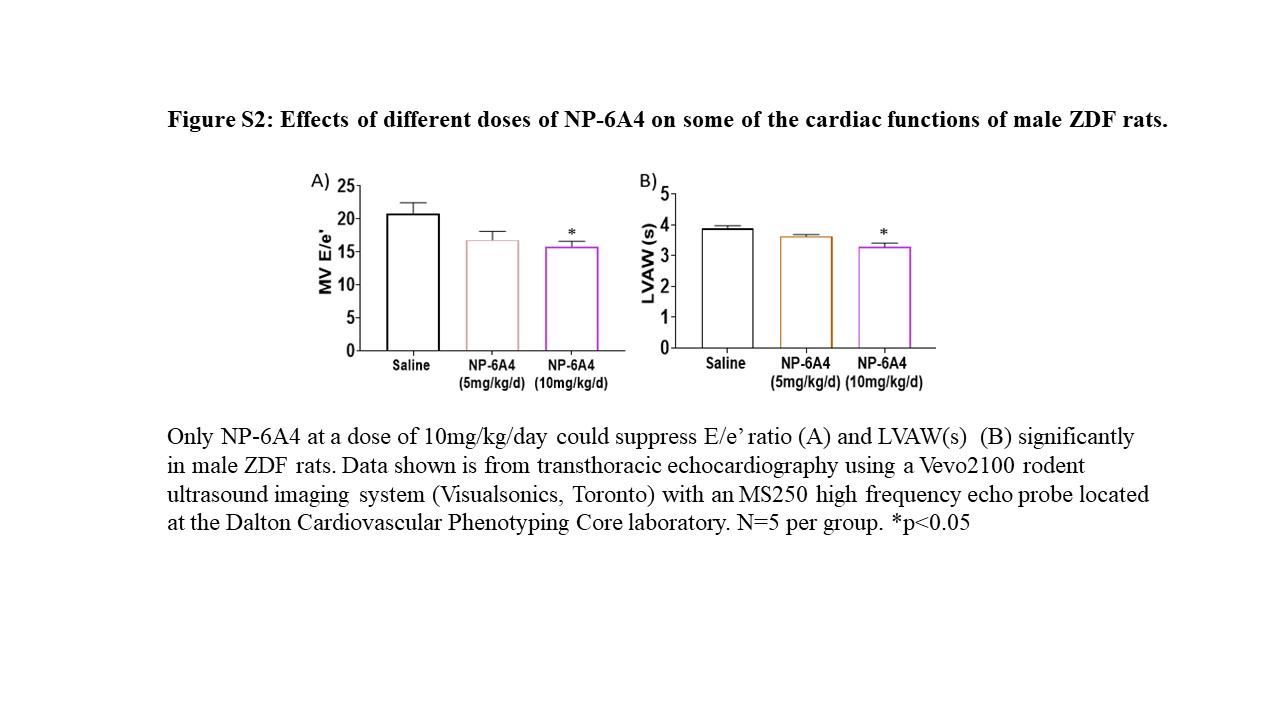

Supplement: Supplementary file 1 [file cells-12-01373-s001.zip › Figure S2.JPG]

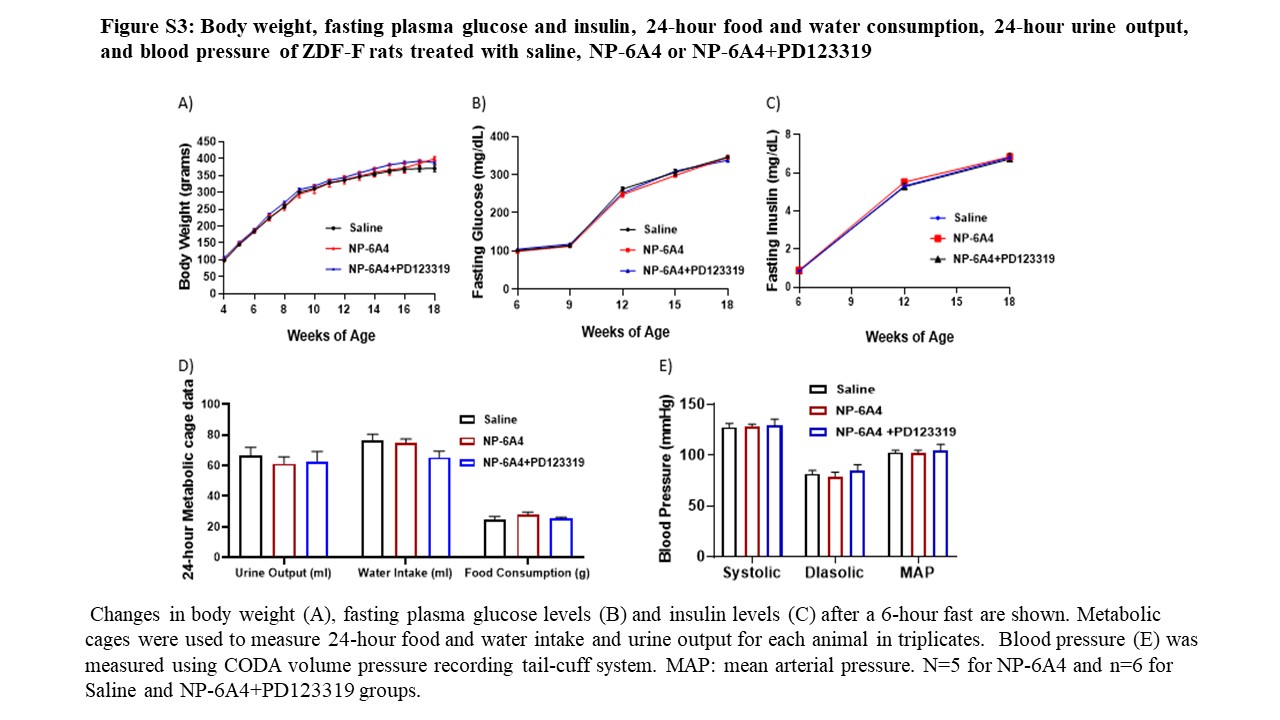

Supplement: Supplementary file 1 [file cells-12-01373-s001.zip › Figure S3.JPG]

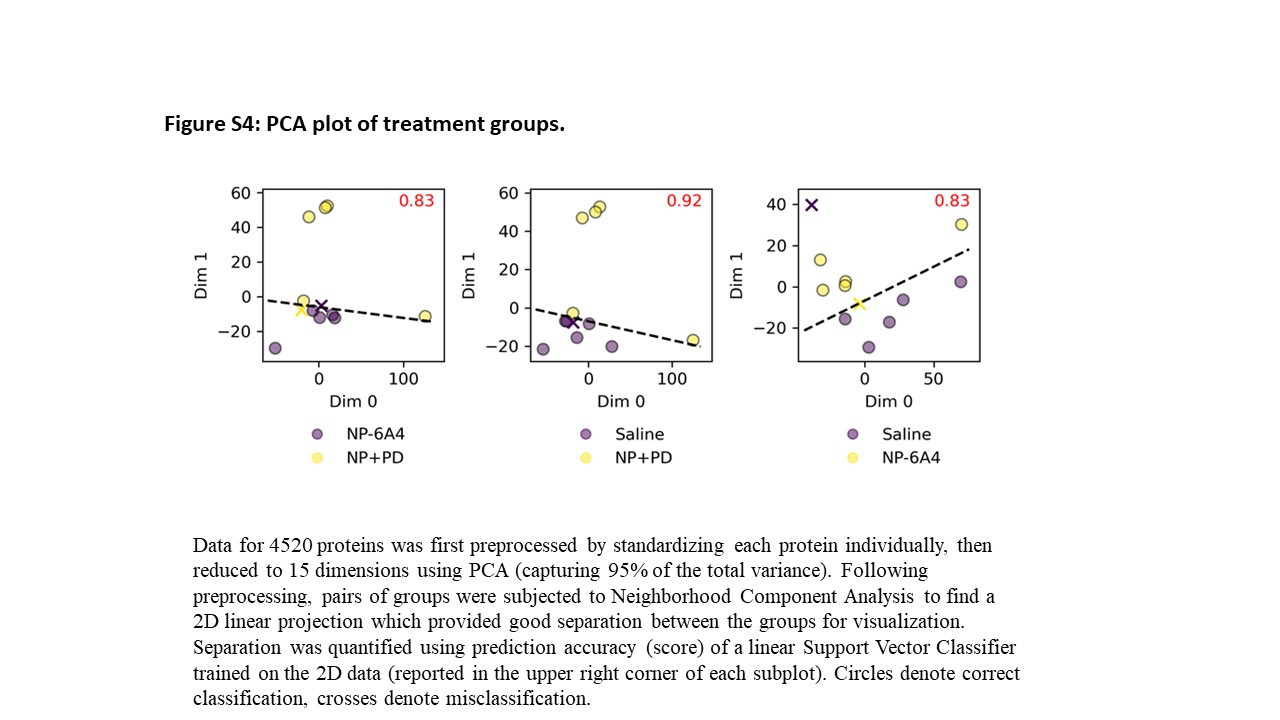

Supplement: Supplementary file 1 [file cells-12-01373-s001.zip › Figure S4.JPG]

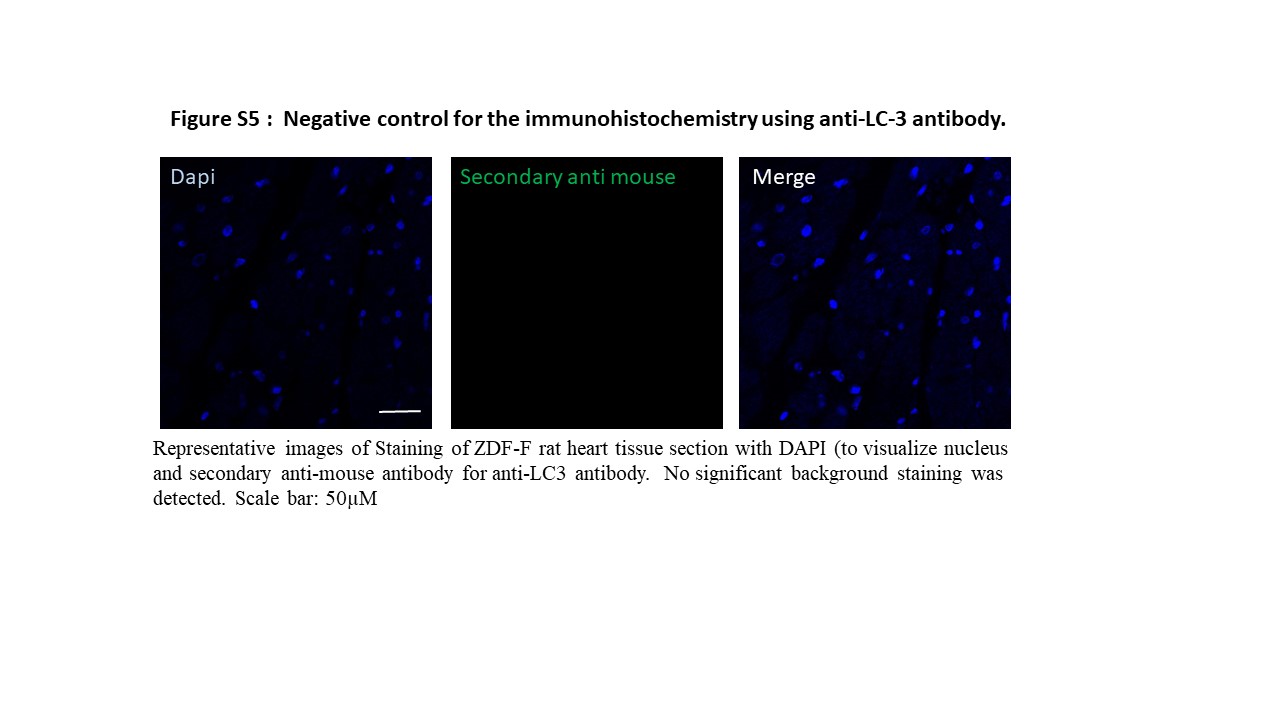

Supplement: Supplementary file 1 [file cells-12-01373-s001.zip › Figure S5.JPG]
